# NINJ1 is activated by cell swelling to regulate plasma membrane permeabilization during regulated necrosis

**DOI:** 10.1038/s41419-023-06284-z

**Published:** 2023-11-18

**Authors:** Yves Dondelinger, Dario Priem, Jon Huyghe, Tom Delanghe, Peter Vandenabeele, Mathieu J. M. Bertrand

**Affiliations:** 1 Inflammation Research Center, VIB, Technologiepark 71, 9052 Zwijnaarde-Ghent, Belgium; 2 Department of Biomedical Molecular Biology, Ghent University, Technologiepark 71, 9052 Zwijnaarde-Ghent, Belgium

**Keywords:** Cell death, Mechanisms of disease, Cell signalling, Inflammation

## Abstract

Plasma membrane permeabilization (PMP) is a defining feature of regulated necrosis. It allows the extracellular release of damage-associated molecular patterns (DAMPs) that trigger sterile inflammation. The pore forming molecules MLKL and GSDMs drive PMP in necroptosis and pyroptosis, respectively, but the process of PMP remains unclear in many other forms of regulated necrosis. Here, we identified NINJ1 as a crucial regulator of PMP and consequent DAMP release during ferroptosis, parthanatos, H_2_O_2_-induced necrosis and secondary necrosis. Importantly, the membrane-permeabilizing function of NINJ1 takes place after the metabolic death of the cells and is independent of the pore-forming molecules MLKL, GSDMD and GSDME. During ferroptosis, NINJ1 acts downstream of lipid peroxidation, which suggested a role for reactive oxygen species (ROS) in NINJ1 activation. Reactive oxygen species were however neither sufficient nor required to trigger NINJ1-dependent PMP. Instead, we found that NINJ1 oligomerization is induced by the swelling of the cell and that its permeabilizing potential still requires an addition, and yet to be discovered, activation mechanism.

## Introduction

The state of the plasma membrane during cell death determines the inflammatory potential of the dying cell. Indeed, the rupture of the plasma membrane results in the extracellular release of damage-associated molecular patterns (DAMPs), that can activate inflammatory signaling pathways by binding to plasma membrane receptors on the surface of innate immune cells [1, 2]. Consequently, necrotic cells are potent inducers of sterile inflammation. This situation contrasts with apoptosis, a cellular suicide program during which the plasma membrane remains intact until the phagocytic removal of the dying cell [3]. Nevertheless, in absence of sufficient phagocytic capacity, apoptotic cells lose their plasma membrane integrity over time in a process called secondary necrosis, and consequently also acquire inflammatory potential [4]. Over the last decades, various forms of regulated necrosis have been identified and, together with secondary necrosis, have been implicated in the pathogenesis of many inflammatory disorders, including ischemia–reperfusion injury, neurological disorders, systemic lupus erythematosus and atherosclerosis [1, 2, 5]. Preventing DAMP release from necrotic cells is therefore currently considered as a new therapeutic strategy against inflammatory diseases [6].

Although our knowledge of the signaling pathways leading to the various forms of regulated necrosis is vastly increasing, our understanding of the process of plasma membrane permeabilization (PMP) is still fragmentary, and in some cases completely unknown [5, 7]. During necroptosis, the phosphorylation-dependent activation of the pseudo-kinase MLKL by RIPK3 has been proposed to induce a pro-death conformational switch in MLKL [8, 9], which subsequently translocates to the plasma membrane to initiate PMP [10, 11]. Similarly, the proteolytic activation of the pore-forming gasdermin D (GSDMD) and E (GSDME) by caspase-1/-8 and -3 is reported to drive PMP during pyroptosis and secondary necrosis, respectively [12–17]. Moreover, the cell surface protein ninjurin-1 (NINJ1) was shown to contribute to PMP during these forms of MLKL/GSDMs-dependent necrosis [18], but it remains unclear how NINJ1 is activated or promotes plasma membrane rupture. In contrast, much less is known on the process of PMP during other forms of regulated necrosis, such as ferroptosis, parthanatos, cuproptosis and H_2_O_2_-induced necrosis.

Ferroptosis is an iron-dependent form of necrosis driven by uncontrolled lipid peroxidation [19]. In healthy cells, lipid peroxidation is negatively regulated by the system X_c_^−^-glutathione-GPX4 axis that cooperates to convert reactive PUFA phospholipid hydroperoxides to non-cytotoxic PUFA phospholipid alcohols [20–23]. Disturbing this main detoxification pathway, either at the level of glutathione import and metabolism, or at the level of GPX4 activity, induces lipid peroxidation and ferroptosis. While our understanding of the regulatory mechanisms determining ferroptosis sensitivity are rapidly increasing, the exact PMP mechanism of ferroptosis remains unknown [20, 21]. Indeed, extensive lipid peroxidation is currently the most downstream step identified, but it is unclear how this process results in PMP [24]. Parthanatos is instead driven by hyperactivation of the DNA repair enzyme poly(ADP-ribose) polymerase-1 (PARP-1) [25, 26]. Extensive DNA damage, induced by oxidative stress, ionizing radiation or DNA alkylation, results in uncontrolled PARP-1 activity, which then promotes the formation and subsequent nuclear translocation of an AIF-MIF protein complex [27–30]. Nuclear MIF will then utilize its nuclease activity to induce chromatinolysis [30]. How these signaling events finally result in PMP is unknown.

In this study, we identified NINJ1 as a crucial mediator of PMP and DAMP release during certain forms of ferroptosis, parthanatos and H_2_O_2_-induced necrosis.

## Results

### NINJ1 regulates plasma membrane permeabilization during secondary necrosis, ferroptosis, parthanatos, cuproptosis and H_2_O_2_-induced necrosis, but not necroptosis

**Fig. 1:**
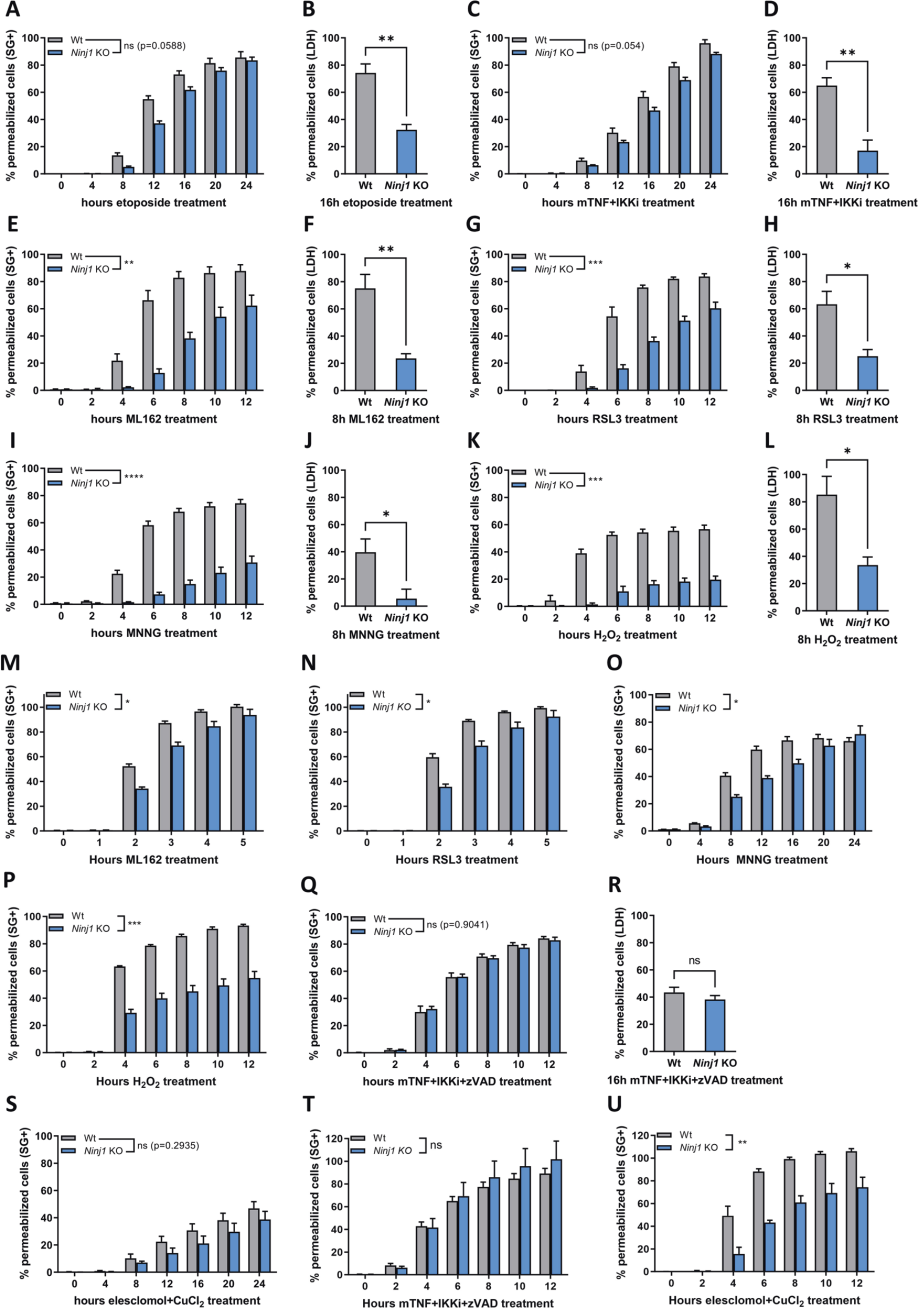
NINJ1 regulates plasma membrane permeabilization during secondary necrosis, ferroptosis, parthanatos, cuproptosis and H_2_O_2_-induced necrosis, but not necroptosis. Wild-type and *Ninj1* KO MEFs (**A**–**L**, **Q**–**S**) and RAW264.7 murine macrophage-like cells (**M**–**P**, **T**, **U**) were stimulated with the indicated compounds. PMP was either measured in function of time by Sytox Green positivity (**A**, **C**, **E**, **G**, **I**, **K**, **M**–**Q**, **S**–**U**) or at the indicated timepoint by LDH release (**B**, **D**, **F**, **H**, **J**, **L**, **R**). Data in the graphs are presented as mean ± SEM of independent experiments (*n* = 3). Statistical significance was determined by two-way ANOVA for Sytox Green positivity or by an unpaired two-tailed *T* test for LDH release. *P ≤ 0.05, **P ≤ 0.01, ***P ≤ 0.001, ****P ≤ 0.0001.

NINJ1 was recently identified as a novel mediator of plasma membrane rupture during pyroptosis, secondary necrosis and necroptosis [18]. The involvement of NINJ1 in all these different signaling pathways may indicate a global role for NINJ1 in PMP [18]. To test whether NINJ1 regulates PMP during additional forms of necrosis, we stimulated wild-type (Wt) and NINJ1 deficient cells with different necrotic triggers and evaluated PMP by Sytox Green positivity or LDH release. We confirmed a role of NINJ1 in PMP during secondary necrosis after an intrinsic or extrinsic apoptotic insult (Fig. 1A–D). NINJ1 deficiency in MEFs significantly reduced LDH release but not Sytox Green positivity, suggesting that NINJ1 only promotes the permeability for large molecules during secondary necrosis, as previously reported during pyroptosis [18]. In contrast, NINJ1 deficiency inhibited both Sytox Green positivity and LDH release after ferroptosis induction by the GPX4 inhibitors ML162 and RSL3 (Fig. 1E–H), after parthanatos induction by the DNA alkylating agent methylnitronitrosoguanidine (MNNG) (Fig. 1I, J), and during H_2_O_2_-induced necrosis (Fig. 1K, L). These findings were validated by PMP assays using Yoyo-1, an alternative small plasma membrane-impermeable DNA binding dye, and by reconstitution of NINJ1 deficient MEFs with wild-type NINJ1 (SFig. 1A–G). These findings indicated that NINJ1 regulates the release of both small and larger molecules depending on the induced cell death modality. Importantly, the role of NINJ1 in PMP during secondary necrosis, ferroptosis and parthanatos was not a consequence of crosstalk between these cell death modalities. Indeed, induction of secondary necrosis, ferroptosis and parthanatos was fully prevented by their respective specific inhibitors, and these inhibitors did not have any protective effects against the other cell death triggers (SFig. 1H–P). H_2_O_2_ can induce different forms of cell death depending on the concentration and cell type exposed [31]. In MEFs, H_2_O_2_-mediated cell death initially involved parthanatos and was followed by another unidentified ROS-dependent cell death modality that was not apoptosis, necroptosis nor ferroptosis (SFig. 1Q–S). Of note, NINJ1-dependent PMP during ferroptosis, parthanatos and H_2_O_2_-induced necrosis was not limited to MEFs but also observed in RAW264.7 murine macrophage-like cells (Fig. 1M–P). Lastly, we tested the contribution of NINJ1 to PMP during necroptosis and cuproptosis, a newly identified form of regulated necrosis triggered by lethal copper levels that lead to lipoylation of TCA enzymes [32]. Interestingly, NINJ1 did not contribute to PMP during necroptosis of MEFs or RAW264.7 cells (Fig. 1Q, R, T and SFig. 1T), but did regulate PMP during cuproptosis of RAW264.7 cells (Fig. 1S, U and SFig. 1U, V). Together, our results demonstrate that NINJ1 promotes PMP during additional but not all forms of regulated necrosis, thereby broadening its implication in cell death.

### NINJ1 promotes DAMP release during NINJ1-dependent necrosis

**Fig. 2:**
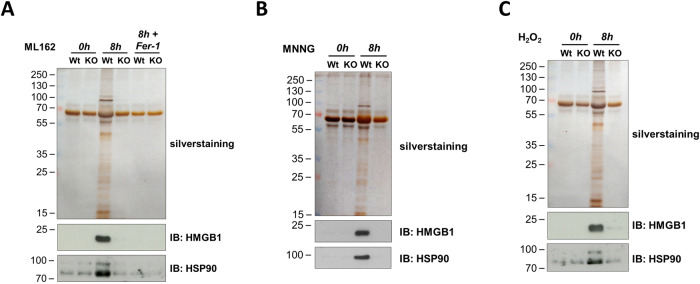
NINJ1 promotes DAMP release during NINJ1-dependent necrosis. Wild-type (Wt) and *Ninj1* KO (KO) MEFs were stimulated for 8 h with ML162 (**A**), MNNG (**B**) or H_2_O_2_ (**C**). TCA-precipitated cell culture supernatants were analyzed by silver staining and immunoblotting. The results are representative of at least two independent experiments.

The role of NINJ1 in regulating the permeability of cells dying by ferroptosis, parthanatos or H_2_O_2_-induced necrosis, prompted us to investigate whether NINJ1 would also regulate the release of DAMPs from these dying cells. After stimulation with ML162 (Fig. 2A), MNNG (Fig. 2B) or H_2_O_2_ (Fig. 2C), we collected the cell-free culture supernatant and precipitated the released proteins with TCA. We observed that NINJ1 promotes the release of various proteins from these necrotic cells, including known DAMPs such as HMGB1 and HSP90 (Fig. 2A–C) [33, 34]. In contrast, NINJ1 deficiency did not impact the release of DAMPs from cells undergoing necroptosis (SFig. 2), a cell death modality in which PMP was found not to rely on NINJ1 (Fig. 1Q, R, T and SFig. 1T).

### NINJ1 acts independently of the known pore-forming proteins MLKL, GSDMD and GSDME

**Fig. 3:**
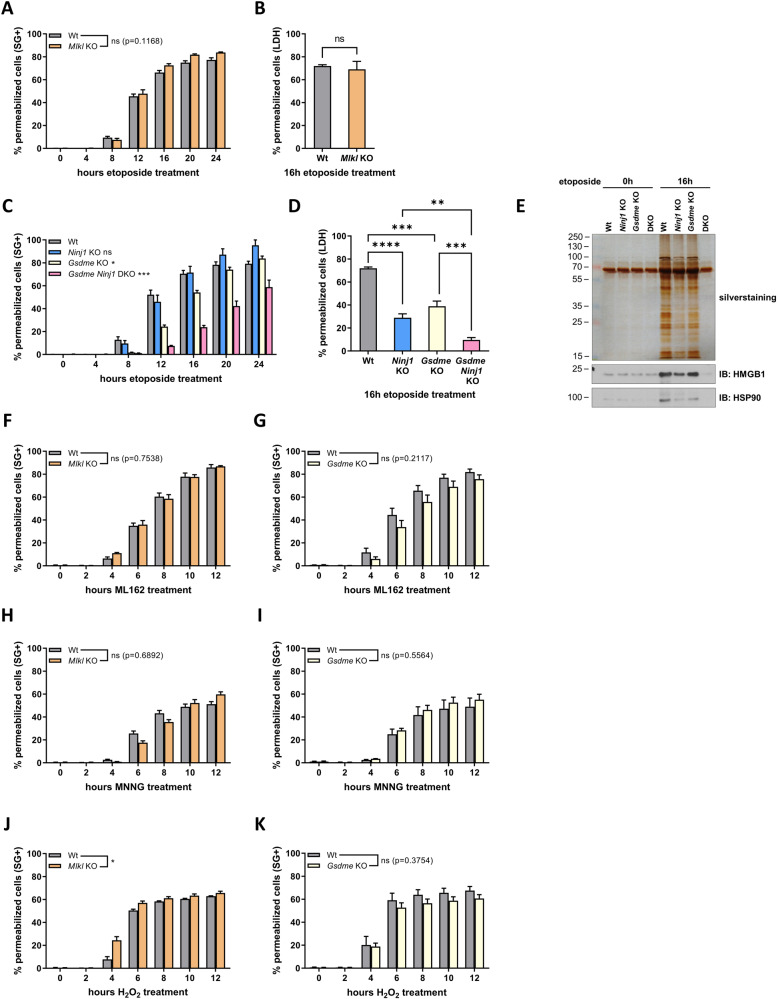
NINJ1 acts independently of the known pore-forming proteins MLKL, GSDMD and GSDME. **A**–**E** Wild-type, *Mlkl* KO, *Ninj1* KO, *Gsdme* KO and *Gsdme Ninj1* DKO (DKO) MEFs were stimulated with etoposide. PMP was measured either by Sytox Green positivity in function of time (**A**, **C**) or by LDH release after 16 h (**B**, **D**). DAMP release was determined by silver staining or immunoblotting after SDS-PAGE of TCA-precipitated cell culture supernatants and is representative of at least two independent experiments (**E**). **F**–**K** Wild-type, *Mlkl* KO and *Gsdme* KO MEFs were stimulated with ML162 (**F**, **G**), MNNG (**H**, **I**) or H_2_O_2_ (**J**, **K**) and PMP was measured by Sytox Green positivity in function of time. Data in the graphs are presented as mean ± SEM of independent experiments (*n* = 3). Statistical significance was determined by an unpaired two-tailed *T* test (**B**), by one-way ANOVA with Tukey post hoc testing (**D**) or by two-way ANOVA (**A**, **C**, **F**–**K**). *P ≤ 0.05, **P ≤ 0.01, ***P ≤ 0.001, ****P ≤ 0.0001.

During pyroptosis of bone marrow-derived macrophages (BMDMs), NINJ1 was proposed to induce PMP downstream of GSDMD pore formation [18]. Activation of NINJ1 by GSDMD can however not be a universal activation mechanism for NINJ1. Indeed, in contrast to BMDMs, MEFs do not express GDSMD, but some forms of regulated necrosis do rely on NINJ1 for PMP (Fig. 1 and SFig. 3A). We therefore hypothesized that NINJ1 activation in our cellular system could instead depend on the activity of the pore forming proteins MLKL and GSDME. In accordance with previous reports, MLKL did not contribute to PMP during secondary necrosis, initially induced by an intrinsic (Fig. 3A, B) or extrinsic (SFig. 3B, C) apoptotic trigger. Instead, PMP was partially dependent on GSDME (Fig. 3C, D and SFig. 3D, F) [14, 15]. The activity of NINJ1 is however independent of GSDME, as double deficiency for GSDME and NINJ1 further delayed PMP (Fig. 3C, D and SFig 3D, F). Accordingly, DAMPs release was also inhibited to a greater extent when both GSDME and NINJ1 are absent in MEFs (Fig. 3E and SFig. 3G). These results indicate that GSDME and NINJ1 have parallel and complementary functions in PMP during secondary necrosis. We found that PMP occurred independent of MLKL or GSDME during NINJ1-dependent ML162-induced ferroptosis, parthanatos and H_2_O_2_-induced necrosis (Fig. 3F–K). Together these results demonstrate that the membrane permeabilizing function of NINJ1 can be dissociated from the activity of the pore-forming proteins MLKL, GSDMD and GSDME.

### NINJ1 regulates plasma membrane permeabilization downstream of metabolic cell death

**Fig. 4:**
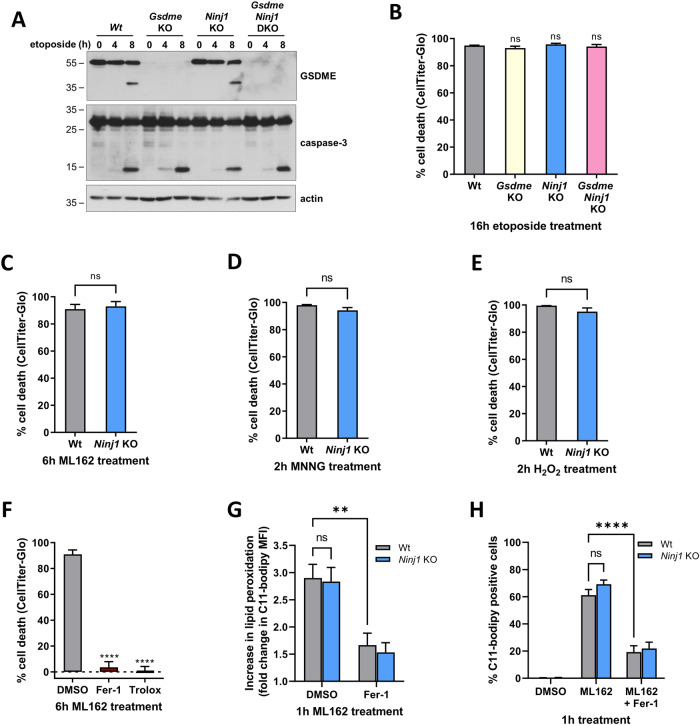
NINJ1 regulates plasma membrane permeabilization downstream of metabolic cell death. **A**, **B** Wild-type, *Gsdme* KO, *Ninj1* KO and *Gsdme Ninj1* DKO MEFs were stimulated with etoposide. Next, the amount of GSDME and caspase-3 processing after the indicated duration was analyzed by immunoblotting (representative of at least two independent experiments) (**A**) or the intracellular ATP levels were measured after 16 h by CellTiter-Glo (**B**). **C**–**E** Wild-type and *Ninj1* KO MEFs were stimulated for 6 h with ML162 (**C**), for 2 h with MNNG (**D**) or for 2 h with H_2_O_2_ before measurement of the intracellular ATP levels by CellTiter-Glo. **F** Wild-type MEFs were pretreated for 30 min with DMSO, Fer-1 or Trolox before stimulation of 6 h with ML162. The intracellular ATP levels were afterwards measured by CellTiter-Glo. **G**, **H** Wild-type and *Ninj1* KO MEFs were pretreated or not for 30 min with Fer-1 before stimulation with ML162 for 1 h. The extent of lipid peroxidation was determined by the fluorescence of oxidized C11-bodipy measured via flow cytometry. Data are presented as the fold change of the MFI over DMSO-stimulated cells (**G**) or as the percentage of C11-bodipy positive cells (**H**). Data in the graphs are presented as mean ± SEM of independent experiments (*n* = 3). Statistical significance was determined by an unpaired two-tailed *T* test (**C**–**E**), by one-way ANOVA with Dunnett post hoc testing (**B**, **F**) or by two-way ANOVA with Sidak post hoc testing. **P ≤ 0.01, ****P ≤ 0.0001.

We next investigated at which stage of the signaling pathways of apoptosis-driven secondary necrosis, ferroptosis, parthanatos and H_2_O_2_-induced necrosis NINJ1 plays a role. While PMP is clearly reduced after 16 hours of etoposide treatment in cells deficient for NINJ1 or for GSDME and NINJ1 (Fig. 3C, D), activation of caspase-3 occurred at the same time and to the same extent in all genotypes (Fig. 4A). These results indicated a role for NINJ1 and GSDME downstream of caspase-3 activation. They consequently specifically contribute to the secondary necrotic phase of this cell death modality. Moreover, the fact that all cell lines died metabolically at the same time point, as measured by cytosolic ATP content, further confirmed the roles of NINJ1 and GSDME as bona fide executioners of PMP (Fig. 4B). NINJ1 similarly functioned at a very late stage of the cell death processes of ferroptosis, parthanatos and H_2_O_2_-induced necrosis, because metabolic cell death levels are not different between Wt and NINJ1 deficient cells (Fig. 4C–E and SFig 4A). Intriguingly, inhibiting lipid peroxidation, the most downstream step identified in the ferroptotic signaling cascade, by ferrostatin-1 (Fer-1) or by the water-soluble Vitamin E analog and ROS scavenger Trolox, did prevent metabolic cell death (Fig. 4F). These results therefore suggested that NINJ1 functions downstream of lipid peroxidation during ferroptosis. To further evaluate this possibility, we directly measured lipid peroxidation in Wt and NINJ1 deficient MEFs after ML162 stimulation. We observed no difference in either the extent of lipid peroxidation or the number of cells with peroxidized lipids (Fig. 4G, H), confirming that NINJ1 functions downstream of lipid peroxidation during ferroptosis.

### Reactive oxygen species are neither sufficient nor required to induce NINJ1-dependent plasma membrane permeabilization

**Fig. 5:**
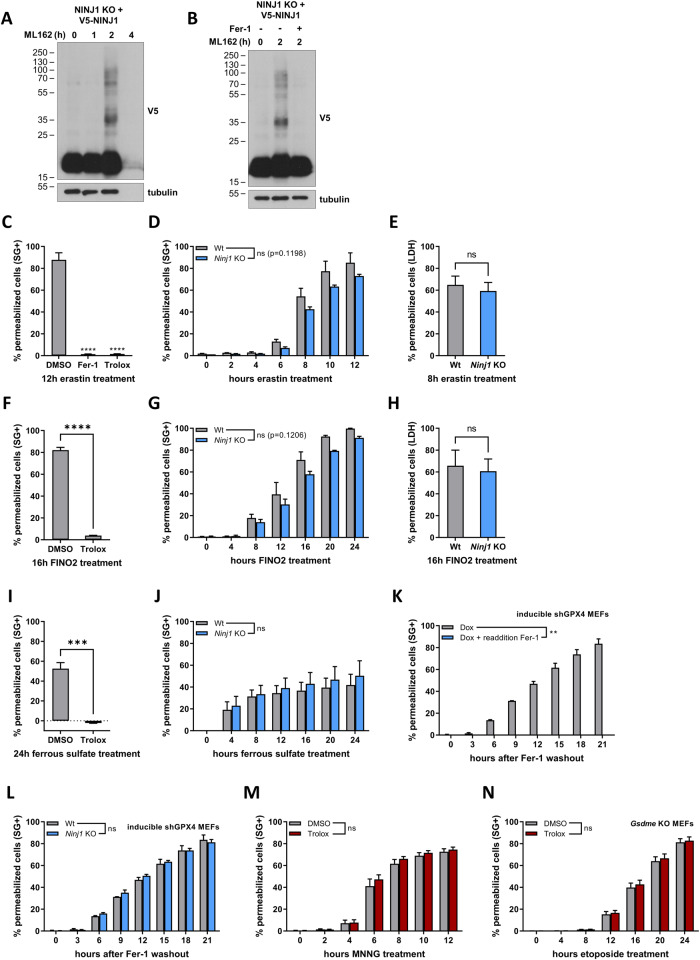
Reactive oxygen species are neither sufficient nor required to induce NINJ1-dependent plasma membrane permeabilization. **A**, **B** V5-NINJ1 reconstituted *Ninj1* KO MEFs were treated with doxycycline overnight and the next day stimulated ML162 for the indicated duration (**A**, **B**) in the presence or absence of 30 min pretreatment with Fer-1 (**B**). NINJ1 oligomerization was determined by immunoblotting after BS³ crosslinking. The results are representative of at least two independent experiments. **C**–**J** Wild-type and *Ninj1* KO MEFs were pretreated for 30 min with DMSO, Fer-1 or Trolox, and then stimulated with the indicated compounds. PMP was determined by Sytox Green positivity in function of time (**C**, **D**, **F**, **G**, **I**, **J**) or by LDH release at the indicated timepoint (**E**, **H**). **K**, **L** Wild-type (**K**, **L**) or *Ninj1* KO MEFs (**L**) expressing an inducible shRNA construct against GPX4 were treated for 48 h with doxycycline in the presence of Fer-1. After 48 h, Fer-1 was either reapplied (**K**) or removed (**K**, **L**) and PMP was determined by Sytox Green positivity in function of time. **M**, **N** Wild-type or *Gsdme* KO MEFs were pretreated for 30 min the presence or absence of Trolox and then stimulated with MNNG (**M**) or etoposide (**N**). PMP was determined by Sytox Green positivity in function of time. Data in the graphs are presented as mean ± SEM of independent experiments (*n* = 3). Statistical significance was determined unpaired two-tailed *T* tests (**E**, **F**, **H**, **I**), one-way ANOVA with Dunnett post hoc testing (**C**) or by two-way ANOVA (**D**, **G**, **J**–**M**, **P**). **P ≤ 0.01, ***P ≤ 0.001, ****P ≤ 0.0001.

Having positioned NINJ1 downstream of lipid peroxidation in the ferroptosis pathway, we next evaluated the possible role of ROS in NINJ1 activation. Indeed, ROS have been associated with NINJ1-dependent cell death forms and administration of H_2_O_2_, a reactive oxygen species, also induces NINJ1-dependent PMP [35–39]. We observed NINJ1 oligomerization, a readout for NINJ1 activity [18, 40], during ML162-induced ferroptosis, parthanatos and H_2_O_2_-induced necrosis (Fig. 5A and SFig 5A, B). Remarkably, NINJ1 oligomerization was prevented by the inhibition of lipid peroxidation, suggesting a casual effect between ROS generation and NINJ1 activation (Fig. 5B). To further explore this possibility, we used several additional ferroptotic triggers including erastin (an inhibitor of the cystine-glumatate antiporter X_c_^−^), FINO2 (an iron oxidizer and an indirect inhibitor of GPX4), ferrous sulfate (increases the intracellular labile iron pool) and by inducible GPX4 knockdown [41–43] (SFig. 5C). While all these triggers result in ROS-dependent cell death, NINJ1 was surprisingly not required for PMP, which was measured by Sytox Green positivity and LDH release (Fig. 5C–L). These results therefore demonstrated that the generation of ROS production is not sufficient to activate NINJ1. Furthermore, MNNG-induced parthanatos and etoposide-induced secondary necrosis in the GSDME deficient MEFs both induce NINJ1-dependent PMP, yet they do not rely on ROS (Fig. 5M, N). Together, these results excluded a general role for ROS in NINJ1 activation.

### NINJ1 is activated by cell swelling

**Fig. 6:**
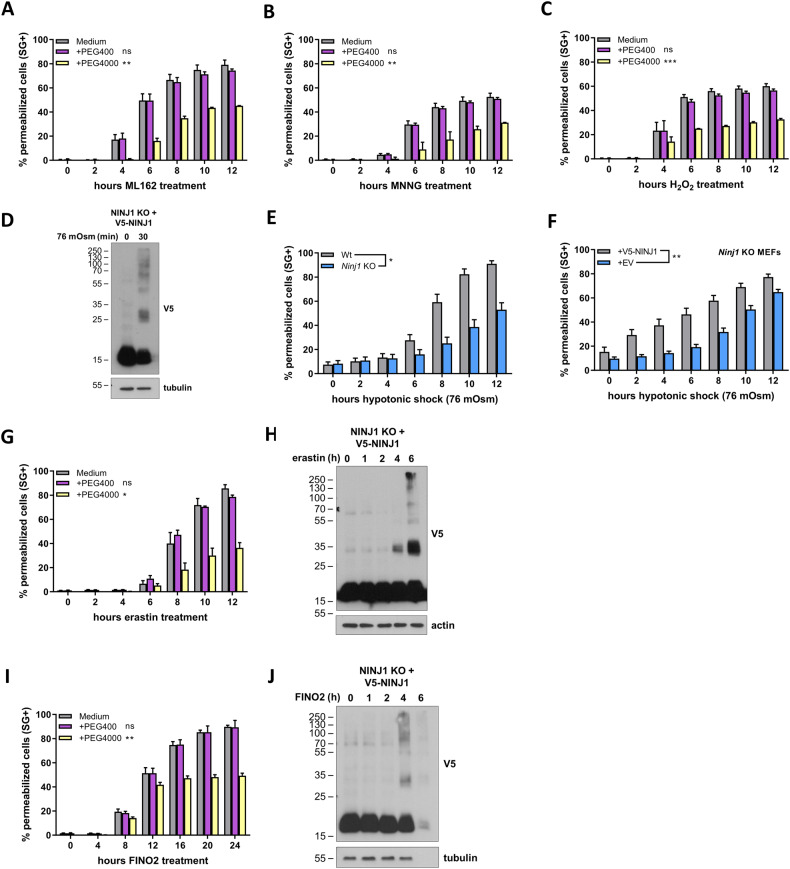
NINJ1 is activated by cell swelling. **A**–**C** Wild-type MEFs were stimulated with ML162 (**A**), MNNG (**B**) or H_2_O_2_ (**C**) in the presence or absence of PEG400 or PEG4000. **D**, **F** V5-NINJ1 reconstituted *Ninj*1 KO MEFs were treated with doxycycline overnight and the next day exposed to a 76 mOsm hypotonic shock. **E** Wild-type and *Ninj1* KO MEFs were exposed to a 76 mOsm hypotonic shock. **G**, **I** Wild-type MEFs were stimulated with erastin (**G**) or FINO2 (**I**) in the presence or absence of PEG400 or PEG4000. **H**, **J** V5-NINJ1 reconstituted *Ninj*1 KO MEFs were treated with doxycycline overnight and stimulated with erastin (**H**) or FINO2 (**J**) on the next day. Plasma membrane permeabilization in function of time was measured by Sytox Green positivity (**A**–**E**, **G**). NINJ1 oligomerization was determined by immunoblotting after BS³ crosslinking and is representative of at least two independent experiments (**D**, **H**, **J**). Data in the graphs are presented as mean ± SEM of independent experiments (*n* = 3). Statistical significance was determined by two-way ANOVA. *P ≤ 0.05, **P ≤ 0.01, ****P ≤ 0.0001.

Oncosis caused by small membrane pores is one of the final stages of ferroptosis [44, 45]. The exogenous addition of polyethylene glycols (PEG) of sufficient size as osmoprotectants can counteract the activity of these pores and greatly delays PMP (Fig. 6A) [44, 45]. While oncosis is an established feature of ferroptosis, it is currently not clear if this process also contributes to parthanatos and H_2_O_2_-induced necrosis. We found that the addition PEG4000 (hydrated radius: 1.8 nm) similarly delayed PMP after MNNG and H_2_O_2_ stimulation, a protection that was not observed with PEG400, a PEG of smaller size (hydrated radius: 0.56 nm) used as control (Fig. 6B, C). Consistent with a role of NINJ1 downstream of metabolic cell death (Fig. 4B–E), PEG4000 did not protect the cells against metabolic death during parthanatos or H_2_O_2_-induced necrosis (SFig. 6A, B). Surprisingly, PEG4000 (and PEG400) did provide partial protection against metabolic cell death during ML162 induced ferroptosis (SFig. 6C), which may indicate that it interferes with an earlier, NINJ1-independent, signaling step during this specific form of necrosis.

Having established that the NINJ1-driven cell death modalities are characterized by oncosis, we next determined if cell swelling could be sufficient to trigger NINJ1 activation. To do so, we exposed wild-type and NINJ1 deficient MEFs to a hypotonic shock by reducing the osmolarity of the medium from 360 mOsm to 76 mOsm. Remarkably, hypotonicity sufficed to induce NINJ1 oligomerization and NINJ1-dependent PMP (Fig. 6D–F). Of note, ferroptosis induced by FINO2 and erastin also resulted in oncosis and consequently NINJ1 oligomerization (Fig. 6G–J). Since our previous results demonstrated that PMP does not rely on NINJ1 following stimulation of the cells with these ferroptotic triggers (Fig. 4F, I), these results indicate that NINJ1 oligomerization does not necessarily result in NINJ1-dependent PMP.

## Discussion

The process of PMP has remained obscure for many forms of regulated necrosis. Our results now provide some insights on how PMP is regulated during ferroptosis induced by GPX4 inhibition, parthanatos and H_2_O_2_-induced necrosis. We demonstrate that these necrotic cell death modalities do not rely on the pore forming molecules MLKL and GSDME but require NINJ1 for their PMP. During these cell death forms, NINJ1 oligomerizes and promotes the permeabilization of metabolically dead cells. NINJ1 was previously identified as an executioner of plasma membrane rupture during secondary necrosis, pyroptosis and necroptosis of BMDMs [18]. Implication of NINJ1 to all these forms of regulated necrosis could suggest a global role of NINJ1 in PMP. However, our results indicate that the contribution of NINJ1 to PMP is cell- and trigger-dependent. Indeed, contrary to previous findings in BMDMs [18], we found that PMP occurs independently of NINJ1 during necroptosis in MEFs and RAW264.7 cells. During cuproptosis, it relied on NINJ1 in RAW264.7 cells but not in MEFs. Furthermore, we found that the requirement of NINJ1 for PMP during ferroptosis is trigger dependent. It requires NINJ1 when ferroptosis is induced by RSL3 or ML162, but not by FINO2 or erastin. These results contrast with a recent study excluding a role for NINJ1 in PMP during ferroptosis induced by RSL3 in ASC-overexpressing RAW264.7 cells [46]. The reason for this cell- and trigger-dependent function of NINJ1 in PMP is currently unknown. It is also unclear why NINJ1 controls PMP in many but not all forms of necrosis. A potential explanation could reside in the existence of multiple parallel pathways of PMP. Accordingly, the role of NINJ1 would only be revealed when the redundant pathways are not active, which could be trigger- and cell type-dependent. The idea of parallel pathways of PMP is supported by the fact that we only observed a role for NINJ1 in PMP (measured by Sytox Green positivity) during etoposide-induced secondary necrosis when GSDME had previously been deleted. This hypothesis could explain the conflicting reports on the implication, or not, of GSDME in secondary necrosis [14, 15, 47–49].

The role of NINJ1 in a broad spectrum of necrotic pathways suggests a simple and common activation mechanism. We positioned the function of NINJ1 downstream of lipid peroxidation during ferroptosis—thereby identifying NINJ1 as the most downstream player in this pathway to date—but excluded a role for ROS in NINJ1 activation. Instead, we found cell swelling to be common trigger of NINJ1 oligomerization in the studied cell death pathways. Oncosis is indeed a classical feature of necrotic cells. Intriguingly, NINJ1 oligomerization did not always correlate with the requirement of NINJ1 in PMP. This apparent discrepancy may be explained by the co-existence of additional pathways of PMP with faster kinetics, as mentioned above. Alternatively, NINJ1 oligomerization may only be an intermediate step in its activation process and additional factors could be required for its full activation. Further research is therefore required to dissect the process of NINJ1 activation downstream of its oligomerization. Interestingly, we found that hypotonicity and hypertonicity equally activate NINJ1 (Fig. 6D–F and SFig. 6D–F) indicating that NINJ1 can sense opposing conditions of membrane tension and cell volume [50].

The physiological relevance of NINJ1-dependent PMP is not completely clear. NINJ1 deficiency increases the susceptibility of mice towards *Yersinia* or *Citrobacter* infection, suggesting that NINJ1-mediated DAMP release from pyroptotic cells promotes microbial clearance [18, 51]. Nevertheless, Wt and NINJ1 deficient mice display equal sensitivity to LPS-induced endotoxemia, another model of pyroptosis-driven inflammatory pathology, thereby questioning the general importance of DAMPs release to an inflammatory phenotype [18, 52]. We found that NINJ1 deficiency reduces the release of DAMPs by cells undergoing ferroptosis induced by GPX4 inhibition, ROS-induced necrosis, parthanatos and secondary necrosis. Further research is now needed to evaluate the potential benefit of NINJ1 deficiency to the severity of inflammatory disorders driven by these cell death modalities.

## Materials and methods

### Antibodies and reagents

The following antibodies were used throughout this manuscript: anti-V5-HRP (Invitrogen, R96125, 1:2000), anti-β-tubulin-HRP (Abcam ab21058, 1:10,000), anti-GSDME (Abcam ab215191, 1:1000), anti-caspase-3 (Cell Signaling #9662, 1:1000), anti-actin (MP Biomedicals #69100, 1:10,000), anti-NINJ1 (ThermoFisher Scientific PA5-95755, 1:1000), anti-HMGB1 (Abcam ab18256, 1:1000) and anti-HSP90 (Abcam ab13492, 1:1000). The following compounds were used: etoposide (100 µM, Selleckchem S1225), IKKi (TPCA-1, 5 µM, Tocris Bioscience), ML162 (1 µM, Aobious Inc. AOB1514), RSL3 (3 µM, Selleckchem S8155), H_2_O_2_ (500 µM, Sigma-Aldrich H1009), MNNG (500 µM, abcr), zVAD-fmk (50 µM, Bachem BACE4026865.0005), Fer-1 (500 nM, Matrix Scientific #053224), Trolox (100 µM, Sigma-Aldrich #238813), PARP-1i (ABT-888/Veliparib, 10 µM, Selleckchem S1004), erastin (10 µM, Selleckchem S7242), FINO2 (30 µM, Sanbio #25096-1) and ferrous sulfate (Ammonium iron(II) sulfate hexahydrate, 500 µM, Santa Cruz #7783-85-9). Recombinant mouse TNF-α, used at 20 ng/ml, was purchased from the VIB Protein Service Facility (Ghent, Belgium).

### Cell lines

Mouse embryonic fibroblasts (MEFs) and RAW264.7 cells (both tested negative for mycoplasma contamination) were cultured in Dulbecco’s modified Eagle’s medium supplemented with 10% fetal calf serum, L-glutamine (2 mM), sodium pyruvate (400 µM), 100 U/ml penicillin and 100 µg/ml streptomycin in normoxic conditions (5% CO_2_).

NINJ1-deficient RAW264.7 cells were received from Prof. Broz (University of Basel). MEFs deficient in NINJ1, GSDME or both proteins simultaneously were generated by CRISPR-mediated genome engineering via the electroporation of Cas9-RNPs. In short, 0.2 nmoles crRNA (IDT) and 0.2 nmoles tracrRNA (#1072532, IDT) were mixed, denatured at 95 °C for 5 min. and re-annealed for 20 min. at room temperature. For double deficiency, a double amount of tracrRNA was used. Alt-R® CRISPR-Cas9 Negative Control crRNA #1 (#1072544, IDT) was used as NT control. Subsequently, 20 μg Cas9-eGFP was added and the mixture was incubated for 10 min. at room temperature. Next, Cas9-RNPs were mixed with 0.2 nmoles Electroporation enhancer (1075915, IDT) and incubated with 1.25 × 10^6^ cells in a total volume of 100 μl Opti-MEM. Finally, cells were electroporated using the NEPA21 electroporator (MEFs: Poring pulse—140 V, length 10 ms, interval 50 ms, 2 pulses, 10% decay, + polarity—Transfer pulse—20 V, length 50 ms, interval 50 ms, 5 pulses, 40% decay, +/−polarity). The next day, GFP-positive cells were sorted using the BD FACSMelody sorter. The following CRISPR guide sequences were used to generate gene-specific knockouts: mNINJ1 (ACTGAGGAGTATGAGCTCAA) and mDFNA5 (CACGGACACCAATGTAGTGC).

### Cell death assays

For all cell death assays (Sytox Green/Yoyo-1 fluorescence, LDH release and CellTiterGlo assays), cells were seeded day before (MEFs: 3333 per well) in duplicates or triplicates in a 96-well plate. The next day, cells were pre-treated with the indicated inhibitors for 30 min and then stimulated with the indicated compounds in 200 µl total volume. For fluorescence-based cell death assays, cells were stimulated in the presence of 5 μM Sytox Green (Invitrogen) or 0.5 µM Yoyo-1 (ThermoFisher). Sytox Green/Yoyo-1 fluorescence intensity was then measured at intervals of one hour using a Fluostar Omega fluorescence plate reader, with an excitation filter of 485 nm and an emission filter of 520 nm, gains set at 1100, 20 flashes per well and orbital averaging with a diameter of 3 mm. For LDH release assays, the 96-well plate was centrifuged for 5 min at 250 × *g* and 50 µl of the resulting cell culture supernatant was analyzed with the CyQUANT™ LDH Cytotoxicity Assay (ThermoFisher) according to the manufacturer’s instructions. For CellTiter-Glo assays, 150 µl cell culture supernatant was removed and 50 µl CellTiter-Glo® 2.0 Reagent was added to the wells. Cell viability was analyzed according to the CellTiter-Glo® 2.0 Assay (Invitrogen) instruction manual. For all cell death assays, the percentage of cell death was calculated as by subtracting the background signal and by using 0.1% Triton-permeabilized cells as a 100% dead population. All cell death data are presented as mean ± SEM of *n* (indicated in the figure) independent experiments.

### Reconstitution of NINJ1-deficient cells and generation of inducible GPX4 knockdown cell lines

The coding sequence for Wt mouse NINJ1 (gBlock, IDT) was fused to the sequences encoding for a V5 epitope and a G4S linker via a polymerase chain reaction with the purpose to generate a V5-G4S linker-NINJ1 fusion construct. The coding sequence of this fusion construct was then cloned into pENTR3C using the GenBuilder™ Cloning Kit (GenScript). The sequence was then recombined into the doxycycline-inducible pSIN-3xHA-TRE-GW destination vector via the LR gateway recombination system (Invitrogen). The cloning of the pLKO.1-puro destination vector expressing a doxycycline-inducible shRNA against GPX4 was described previously [43]. Lentiviral transduction of MEFs was done by standard protocol. Briefly, HEK293T cells were transfected using calcium phosphate with the destination vector in the presence of the lentiviral packaging vectors pMD2-VSVG and psPAX2. The medium was changed after 6 h, and collected 48 h post-transfection. The virus-containing supernatant was then used to infect MEFs. Forty-eight hours after infection, plasmid-containing cells were selected with 2 µg/ml puromycin.

### NINJ1 oligomerization assays

The day before the experiment, 150,000 NINJ1-reconstituted MEFs were seeded in a 6-well plate and subsequently stimulated with 1 µg/ml doxycycline. After stimulation, the cells were washed three times in PBS and incubated with 2.5 mM BS3 (bis(sulfosuccinimidyl)suberate) (ThermoFisher) for 30 min at 4 °C. Afterwards, the cells were washed two times in PBS before lysis in 1× Laemmli buffer and subsequent boiling of the cell lysates. NINJ1 oligomerization was analyzed by immunoblotting.

### Lipid peroxidation

One hundred thousand MEFs were seeded in a 6-well plate the day before the experiment. The next day, cells were pre-treated with the indicated inhibitors for 30 min and then stimulated with the indicated compounds. Cells were then collected by trypsinization, washed in FACS buffer (PBS with 2% FCS) and then stained with 333 nM Sytox Blue (ThermoFisher) and 1 µM BODIPY™ 581/591 C11 for 15 min at 37 °C in FACS buffer. After staining, the cells were washed and the fluorescence intensity of oxidized C11-BODIPY was measured using a BD LSR II flow cytometer in the B525 channel. Sytox Blue-positive cells, detected with the V450 channel, were excluded from the analysis.

### Osmolarity experiments

PEG 400 and PEG 4000 were dissolved in MEF culture media to obtain a final concentration of 10 mM PEG. The PEG-containing medium was subsequently filter-sterilized. For cell death and NINJ1 oligomerization experiments, the culture medium was replaced by PEG-containing culture medium before stimulation when indicated.

Hypotonic culture medium was obtained by diluting normal culture medium (~360 mOsm) with sterile double distilled water. In contrast, hypertonic culture medium was generated by dissolving sorbitol in normal culture medium and subsequent filter sterilization. Before cell death and NINJ1 oligomerization experiments, the culture medium was replaced by hypotonic or hypertonic culture medium when indicated.

### DAMP release

The day before the experiment, 100,000 MEFs were seeded in a 6-well plate. Before stimulation, the medium was changed to Opti-MEM supplemented with 0.1% fetal calf serum, L-glutamine (2 mM), sodium pyruvate (400 µM), 100 U/ml penicillin and 100 µg/ml streptomycin. After the indicated incubation time, the cell culture medium was spun for 5 min at 250 × *g*. 20% TCA was added to the resulting supernatant and this mixture was incubated overnight at 4 °C to allow protein precipitation. The next day, the protein-TCA mixture was centrifuged for 5 min at 20,000 × *g* at 4 °C. The resulting pellet was washed twice in ice-cold 100% acetone and dissolved in Laemmli buffer supplemented with extra Tris.HCl pH 7.5 to ensure neutral pH. Finally, the present proteins were analyzed via immunoblotting or via SDS-PAGE followed by silver staining performed with the Silver Stain Plus Kit (Bio-Rad).

### Statistical analysis

All statistical analyses were performed with Graphpad Prism 9.3. Adequate statistical tests were chosen according to the experimental set-up. Normal distribution was assumed for every statistical test. For every *T* test, an *F*-test was performed to compare variances. Sample sizes were chosen according to the minimal requirements to perform statistical testing.

### Western blots

Uncropped blots of all western blots are provided in the Supplementary File (Uncropped blots).

### Supplementary information

SuppFigures

SupFigure Legends

Uncropped blots

Author list agreement
