# Supplementary figures and images for: NINJ1 is activated by cell swelling to regulate plasma membrane permeabilization during regulated necrosis
Source: Cell Death Dis. 2023 Nov 18;14(11):755. doi: 10.1038/s41419-023-06284-z (PMC10657445; doi:10.1038/s41419-023-06284-z)

# SFigure 1

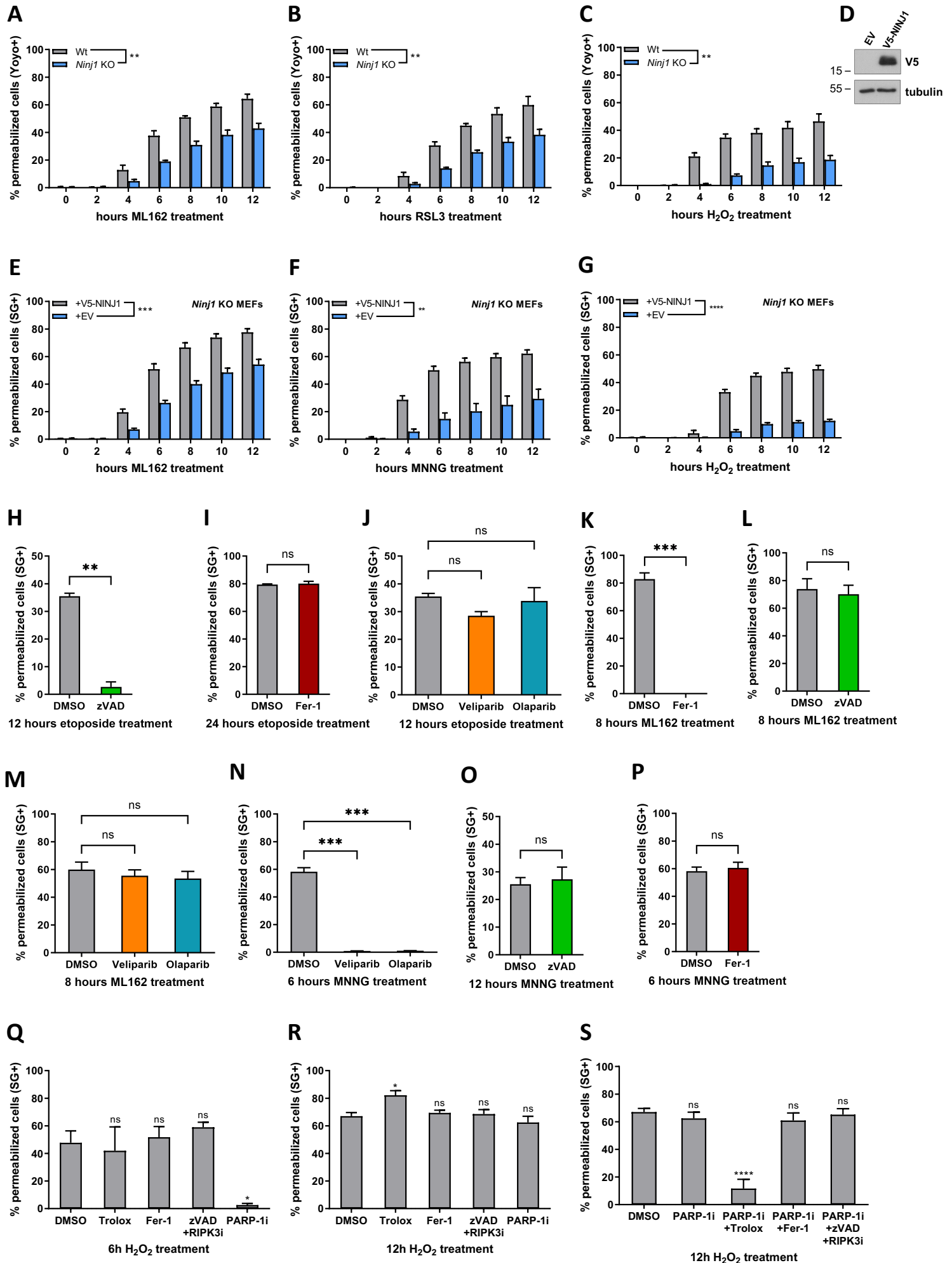

SFigure 1

T

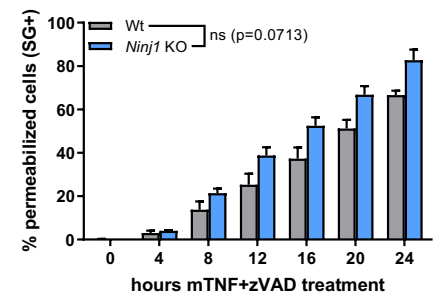

U

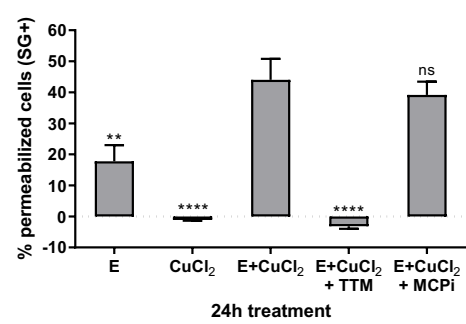

V

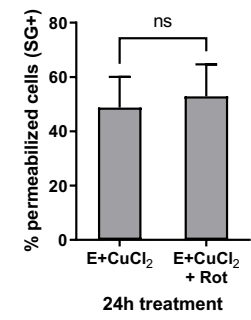

SFigure 2

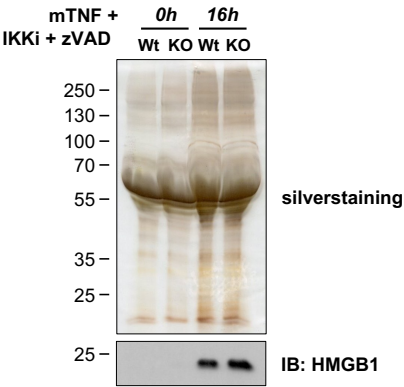

# SFigure 3

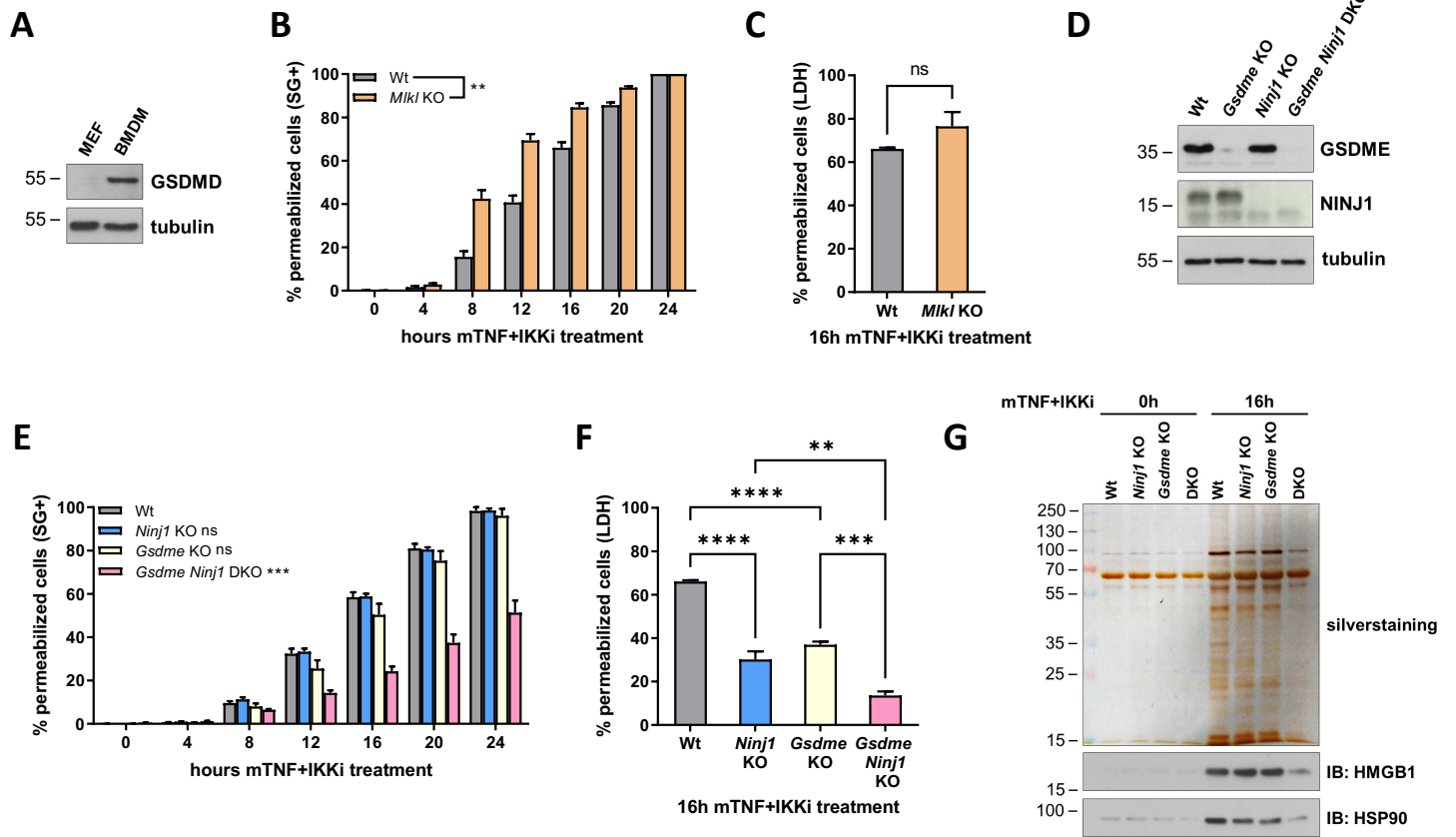

# SFigure 4

A

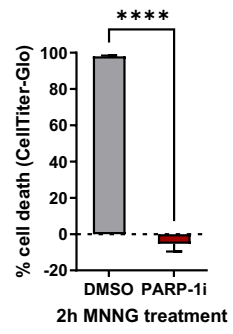

SFigure 5

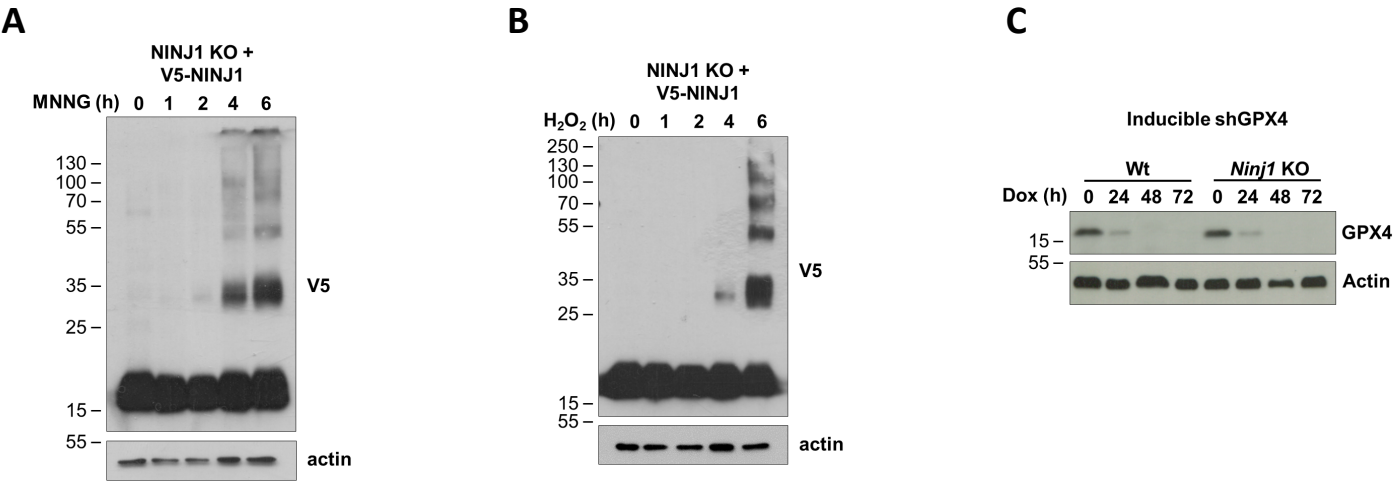

# SFigure 6

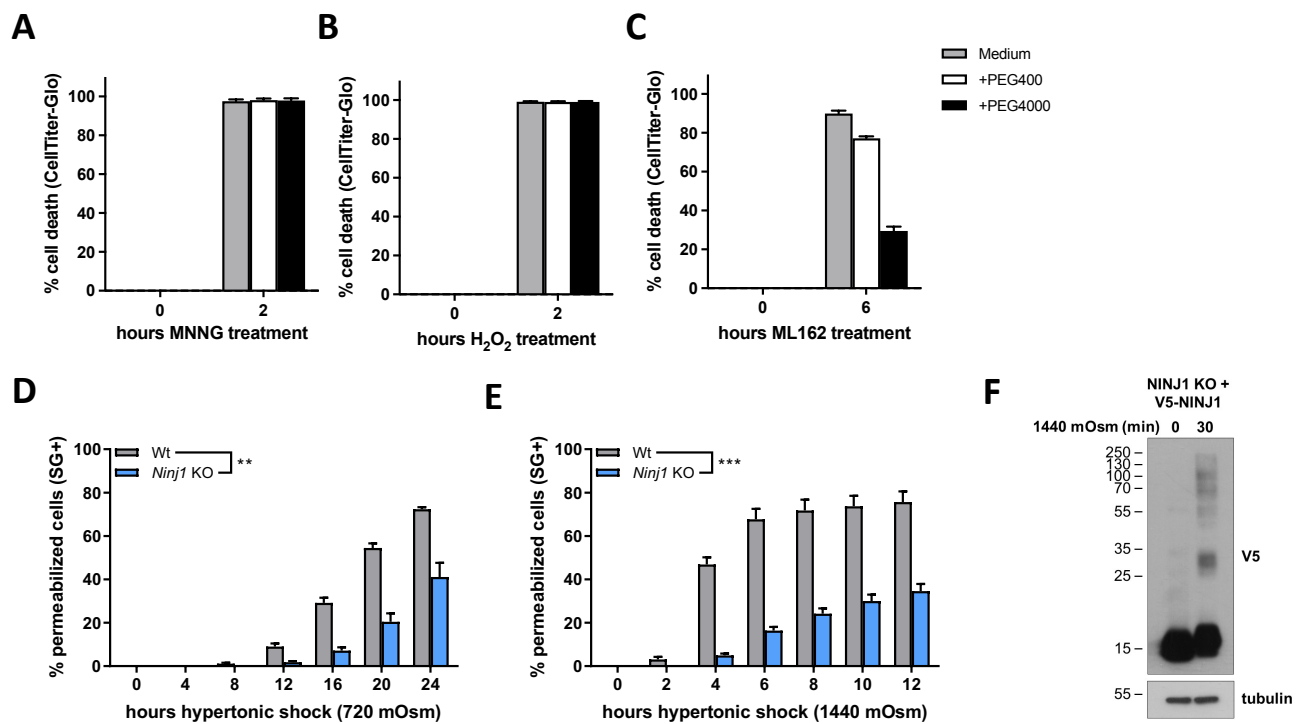

Supplement: Supplementary file 1 — SuppFigures [file 41419_2023_6284_MOESM1_ESM.pdf]
