# Supplementary material for: NINJ1 is activated by cell swelling to regulate plasma membrane permeabilization during regulated necrosis
Source: Cell Death Dis. 2023 Nov 18;14(11):755. doi: 10.1038/s41419-023-06284-z (PMC10657445; doi:10.1038/s41419-023-06284-z)
Supplement: Supplementary file 2 — SupFigure Legends [file 41419_2023_6284_MOESM2_ESM.pdf]

SFig. 1: NINJ1 regulates plasma membrane permeabilization during secondary necrosis, ferroptosis, parthanatos, cuproptosis and H_2O_2-induced necrosis, but not necroptosis. Related to Fig. 1.

(A-C) Wild-type and *Ninj1* KO MEFs were stimulated ML162 (A), RSL3 (B) or H_2O_2 (C). Plasma membrane permeabilization was measured in function of time by Yoyo-1 positivity. (D-G) V5-NINJ1 or empty vector (EV) reconstituted *Ninj1* KO MEFs were treated with doxycycline overnight and stimulated the next day with ML162 (E), MNNG (F) or H_2O_2 (G). (D) Expression levels were determined by immunoblotting. (E-G) Plasma membrane permeabilization was measured in function of time by Sytox Green positivity. (H-S) Wild-type MEFs were pretreated with the indicated inhibitors before stimulation with Etoposide (H-J), ML162 (K-M), MNNG (N-P) and H2O2 (Q-S). (T) Wild-type and *Ninj1* KO MEFs were pretreated with zVAD-fmk (zVAD) for 30min and then stimulated with 20ng/mL mouse TNF. (U-V) Wild-type MEFs were pretreated or not with the Copper chelator Ammonium tetrathiomolybdate (TTM), an inhibitor against the mitochondrial carrier protein (MCPi) or Rotenone (Rot) and then stimulated with elescolomol (E), CuCl2 or the combination of both. Plasma membrane permeabilization was measured by Sytox Green positivity (T-V). Data in the graphs are presented as mean ± SEM of independent experiments (n = 3). Statistical significance was determined by an unpaired two-tailed T-test (H, I, K, L, O, P, V), one-way ANOVA with Dunnett post-hoc testing (J, M, N, Q-S, U) or two-way ANOVA (A-C, E-G, T). * $P \leq 0.05$, ** $P \leq 0.01$, *** $P \leq 0.001$, **** $P \leq 0.0001$.

SFig. 2: NINJ1 promotes DAMP release during NINJ1-dependent necrosis.

Related to Fig. 2.

Wild-type (Wt) and *Ninj1* KO (KO) MEFs were stimulated for 16h with mTNF+IKKi+zVAD. TCA-precipitated cell culture supernatants were analyzed by silverstaining and immunoblotting. The results are representative of two independent experiments.

SFig. 3: NINJ1 acts independently of the known pore-forming proteins MLKL, GSDMD and GSDME. Related to Fig. 3.

(A) GSDMD expression levels of MEFs and bone marrow-derived macrophages were detected by immunoblotting. (B-C) Wild-type and *Mlkl* KO MEFs were pretreated for 30min with an IKK inhibitor and then treated with 20 ng/ml mouse TNF. Plasma membrane permeabilization was measured either by Sytox Green positivity in function of time (B) or by LDH release after 16h (C). (D-G) Wild-type, *Ninj1* KO, *Gsdme* KO and *Gsdme Ninj1* DKO (DKO) MEFs were pretreated for 30 min with an IKK inhibitor and then treated with 20 ng/ml mouse TNF. GSDME and NINJ expression levels were determined by immunoblotting (D). Plasma membrane permeabilization was measured either by Sytox Green positivity in function of time (E) or by LDH release after 16h (F). DAMP release was determined by silver staining or immunoblotting after SDS-PAGE of TCA-precipitated cell culture supernatants and is representative of at least two independent experiments (G). Statistical significance was determined by an unpaired two-tailed T-test (C), by one-way ANOVA with Tukey post-hoc testing (F), by two-way ANOVA for Sytox Green positivity (B, E), * $P \leq 0.05$, ** $P \leq 0.01$, *** $P \leq 0.001$, **** $P \leq 0.0001$.

SFig. 4: NINJ1 regulates plasma membrane permeabilization downstream of metabolic cell death. Related to Fig. 4.

(A) Wild-type MEFs were pretreated for 30 min with a PARP-1 inhibitor and then stimulated for 2h with MNNG. Intracellular ATP levels were measured by CellTiter-Glo. Data in the graphs is presented as mean ± SEM of independent experiments (n = 3). Statistical significance was determined by an unpaired two-tailed T-test. * $P \leq 0.05$, ** $P \leq 0.01$, *** $P \leq 0.001$, **** $P \leq 0.0001$.

SFig. 5: Reactive oxygen species are neither sufficient nor required to induce NINJ1-dependent plasma membrane permeabilization. Related to Fig. 5.

(A-B) V5-NINJ1 reconstituted *Ninj1* KO MEFs were treated with doxycycline overnight and the next day stimulated with MNNG (A) or H_2O_2 (B) for the indicated duration. NINJ1 oligomerization was determined by immunoblotting after BS^3 crosslinking. (C) Wild-type or *Ninj1* KO MEFs expressing an inducible shRNA construct against GPX4 were treated for the indicated duration with doxycycline. GPX4 protein levels were determined by immunoblotting. The results are representative of at least two independent experiments.

SFig. 6: NINJ1 is activated by cell swelling. Related to Fig. 6.

(A-C) Wild-type MEFs were stimulated for 2h with MNNG (A) and H_2O_2 (B) or for 6h with ML162 (C), in the presence or absence of PEG400 or PEG4000, before measurement of the intracellular ATP levels by CellTiter-Glo. Wild-type MEFs (D-F) Wild-type and *Ninj1* KO MEFs were exposed to a 720 mOsm or 1440 mOsm hypertonic shock. (D-E) Plasma membrane permeabilization in function time of was determined by Sytox Green positivity. (F) V5-NINJ1 reconstituted *Ninj*1 KO MEFs were treated with doxycycline overnight and the next day exposed to a 1440 mOsm hypertonic shock for 30 min. NINJ1 oligomerization was determined by immunoblotting after BS^3 crosslinking and is representative of at least two independent experiments. Data in the graphs are presented as mean ± SEM of independent experiments (n = 3). Statistical significance was determined by two-way ANOVA. * $P \leq 0.05$, ** $P \leq 0.01$, *** $P \leq 0.001$, **** $P \leq 0.0001$.
