# Supplementary material for: NINJ1 is activated by cell swelling to regulate plasma membrane permeabilization during regulated necrosis
Source: Cell Death Dis. 2023 Nov 18;14(11):755. doi: 10.1038/s41419-023-06284-z (PMC10657445; doi:10.1038/s41419-023-06284-z)

Fig. 2A

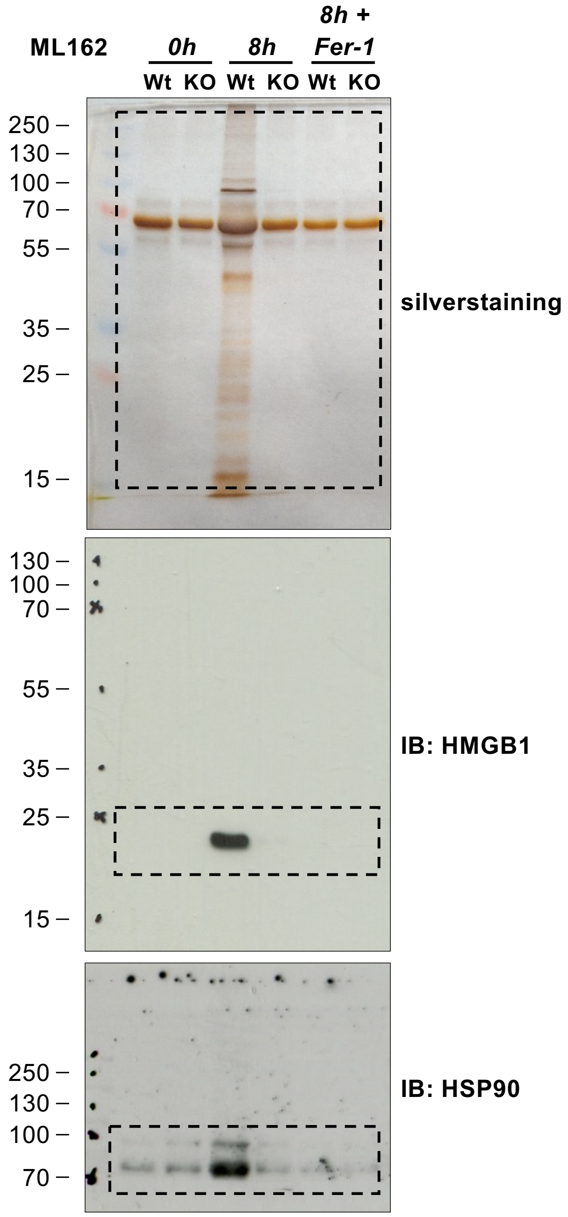
ML162
0h
8h
8h + Fer-1
Wt KO Wt KO Wt KO
250 –
130 –
100 –
70 –
55 –
35 –
25 –
15 –
silverstaining
130 –
100 –
70 –
55 –
35 –
25 –
15 –
IB: HMGB1
250 –
130 –
100 –
70 –
IB: HSP90

Fig. 2B

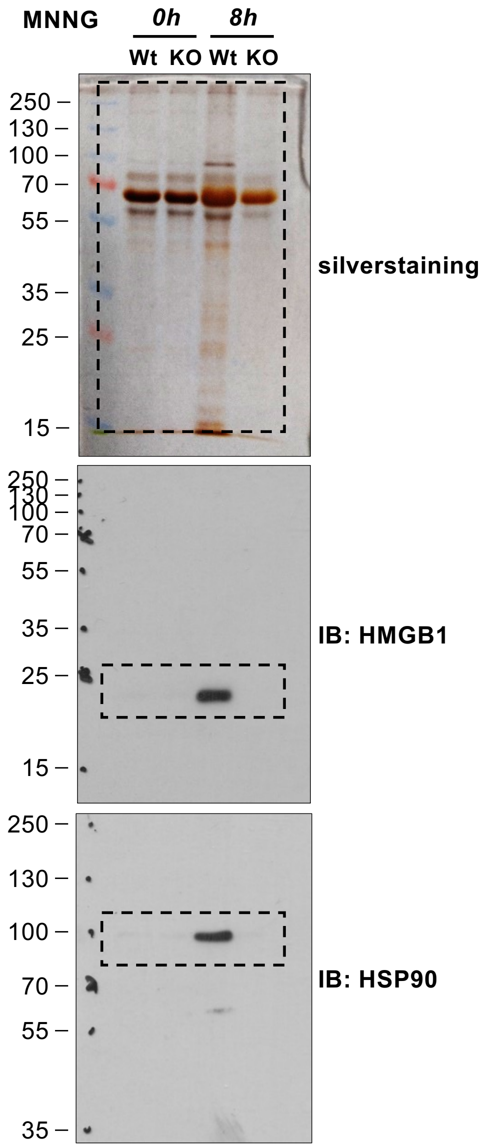
MNNG
0h
8h
Wt KO Wt KO
250 –
130 –
100 –
70 –
55 –
35 –
25 –
15 –
silverstaining
250 –
130 –
100 –
70 –
55 –
35 –
25 –
15 –
IB: HMGB1
250 –
130 –
100 –
70 –
55 –
35 –
IB: HSP90

Fig. 2C

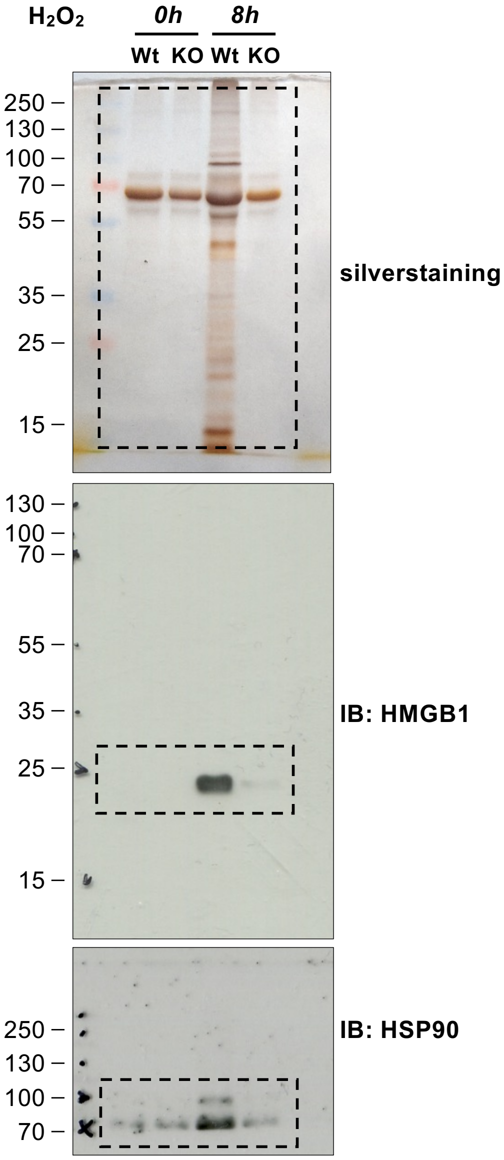

H_2O_2
0h
8h
Wt KO Wt KO
250
130
100
70
55
35
25
15
silverstaining
130
100
70
55
35
25
15
IB: HMGB1
250
130
100
70
IB: HSP90

Fig. 3E

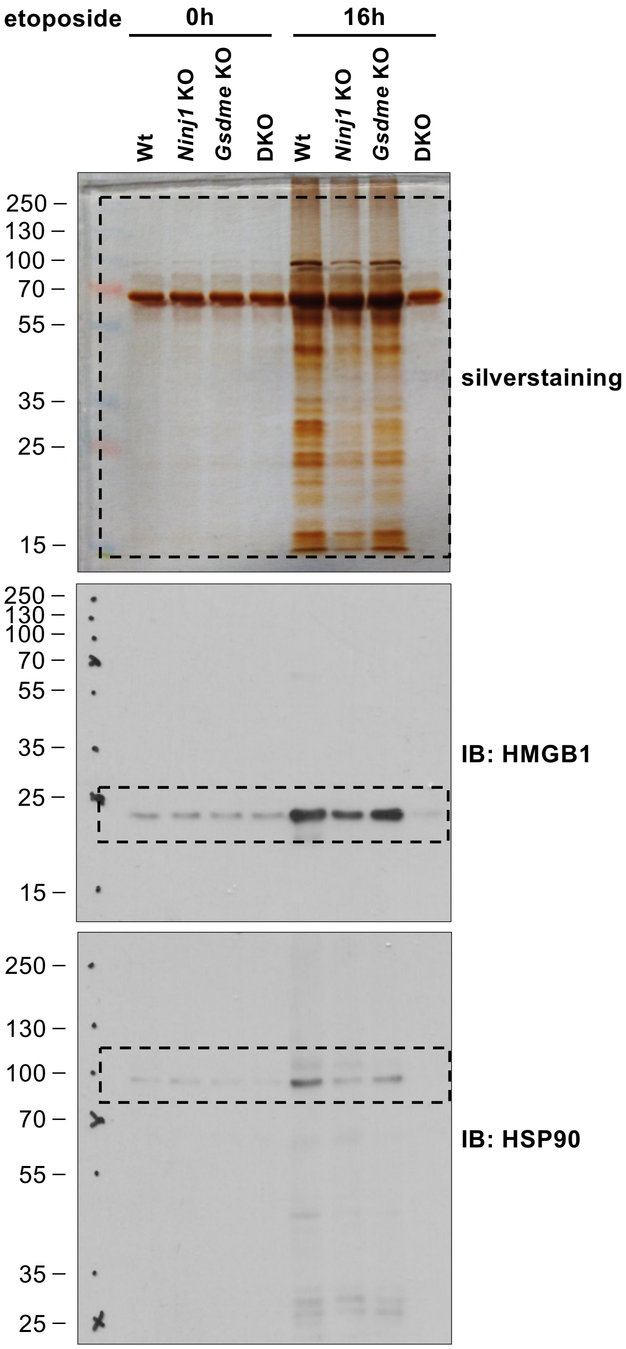

etoposide
0h
16h
Wt
Ninj1 KO
Gsdme KO
DKO
Wt
Ninj1 KO
Gsdme KO
DKO
250
130
100
70
55
35
25
15
silverstaining
250
130
100
70
55
35
25
15
IB: HMGB1
250
130
100
70
55
35
25
IB: HSP90

Fig. 4A

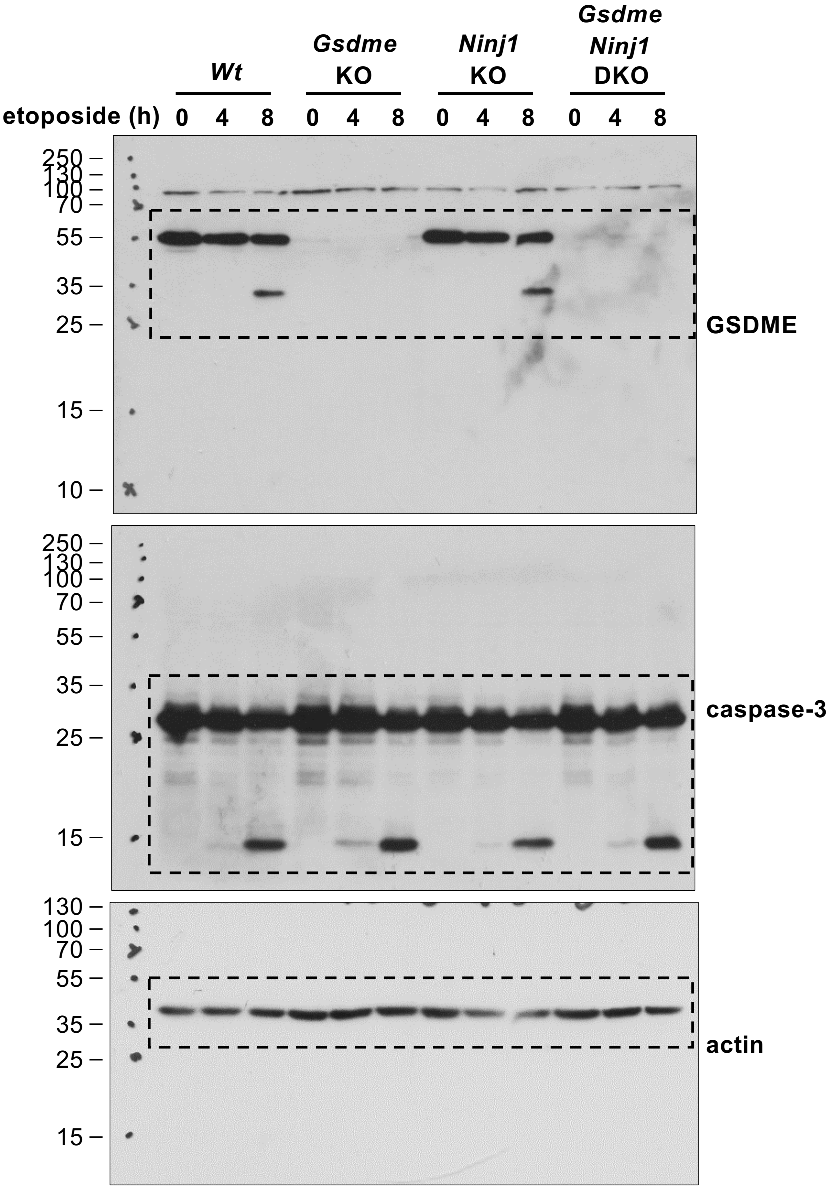
Wt
Gsdme KO
Ninj1 KO
Gsdme Ninj1 DKO
etoposide (h) 0 4 8 0 4 8 0 4 8 0 4 8
250
130
100
70
55
35
25
15
10
GSDME
caspase-3
actin

Fig. 5A

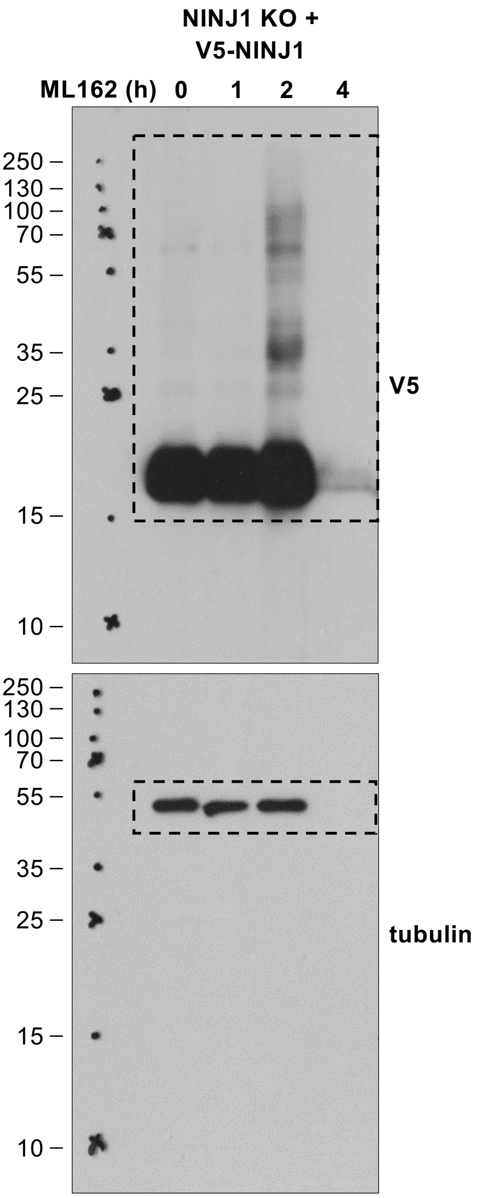
NINJ1 KO + V5-NINJ1
ML162 (h) 0 1 2 4
250
130
100
70
55
35
25
15
10
V5
tubulin

Fig. 5B

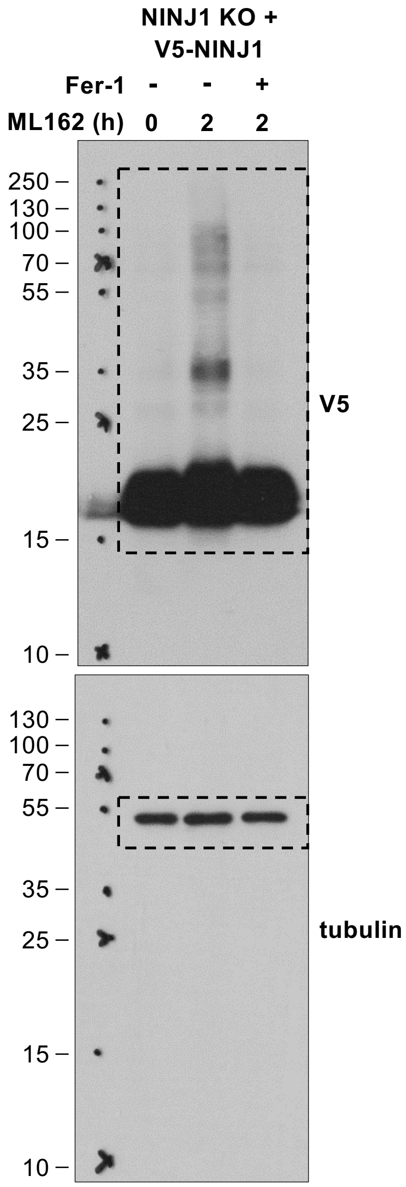

Fig. 6D

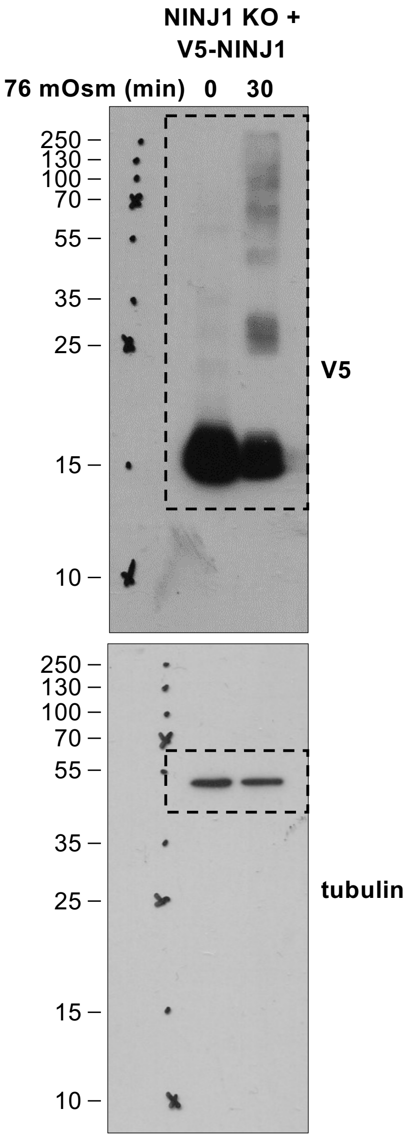

Fig. 6h

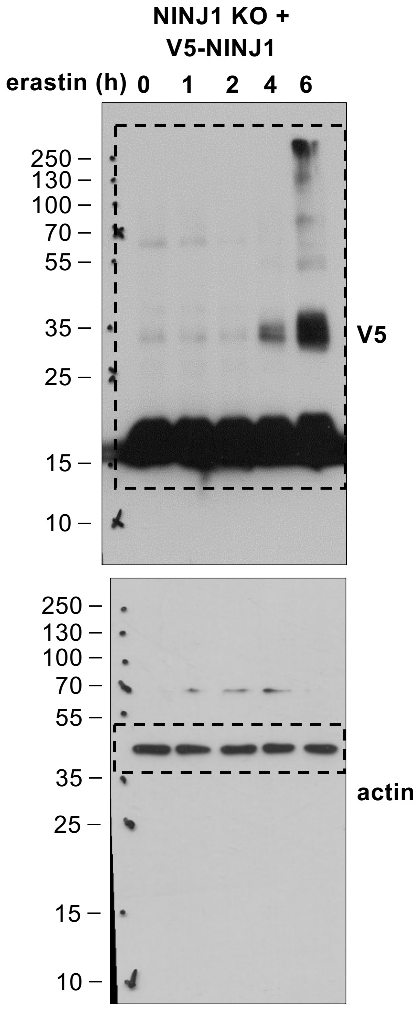

Fig. 6J

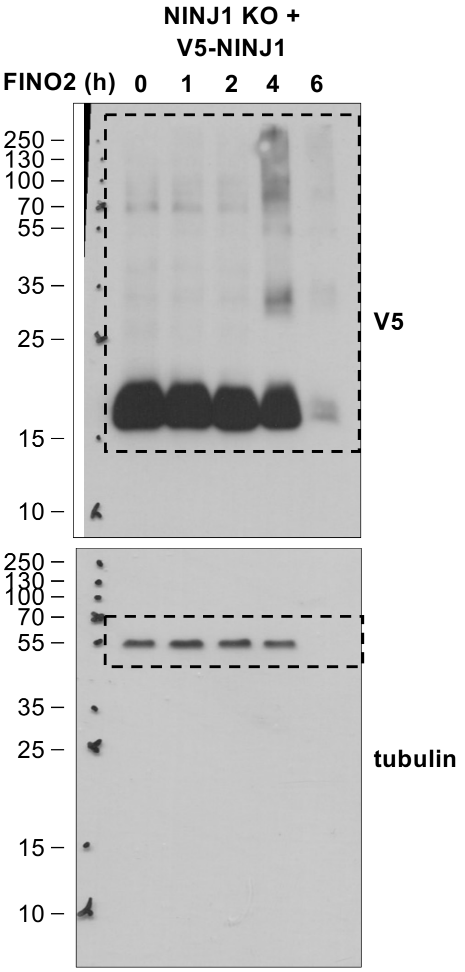

SFig. 1D

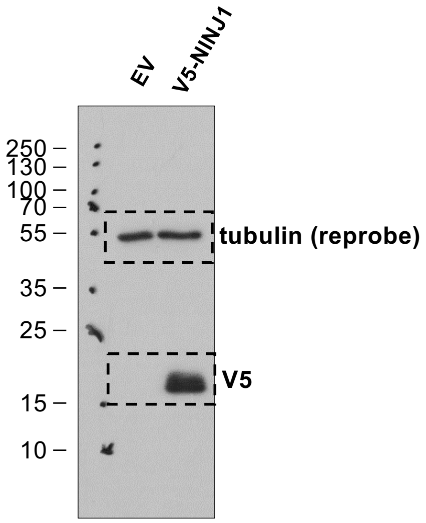

SFig. 3A

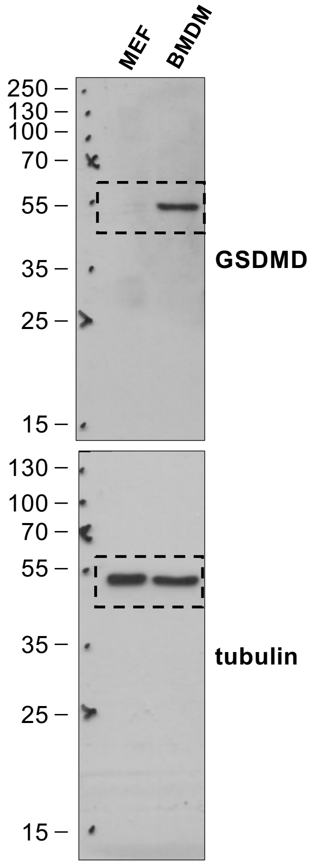

SFig. 2

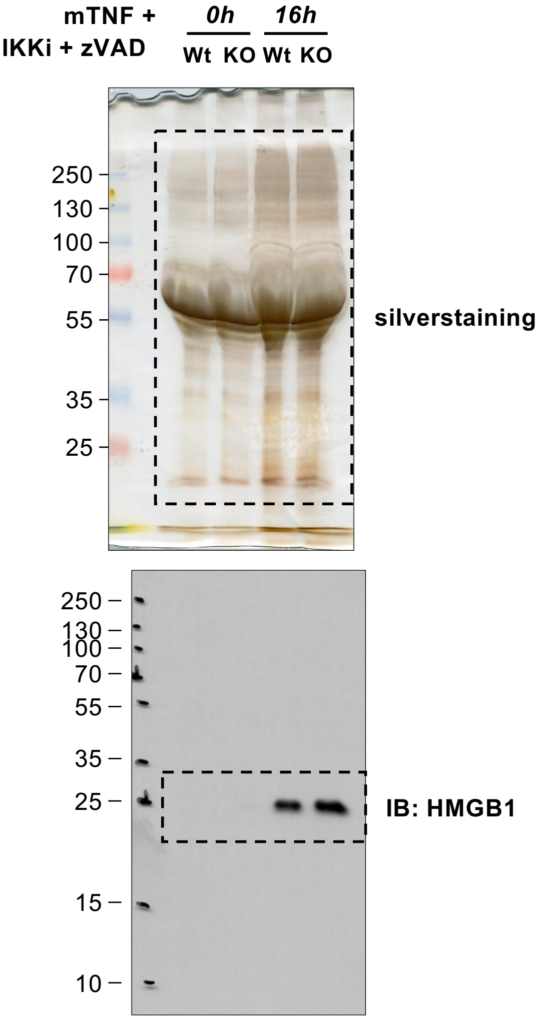

SFig. 3D

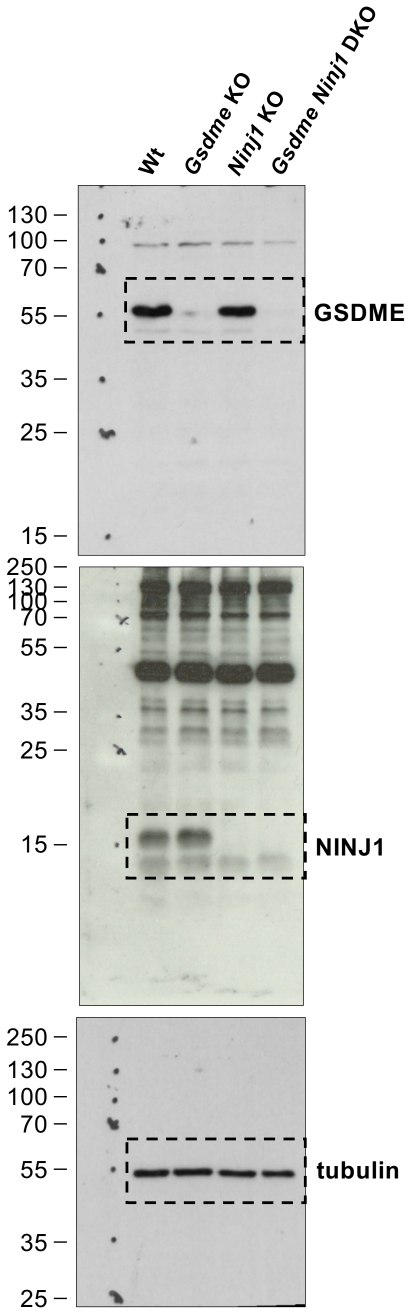

SFig. 3G

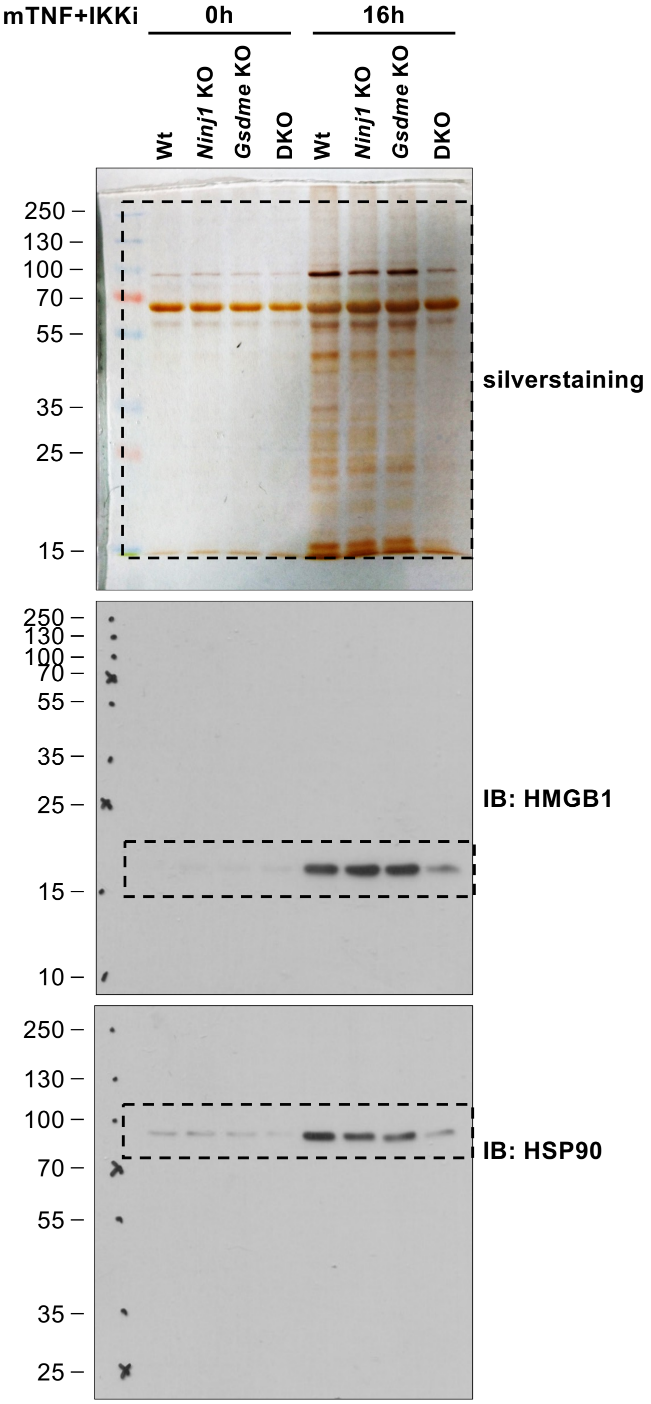

SFig. 5A

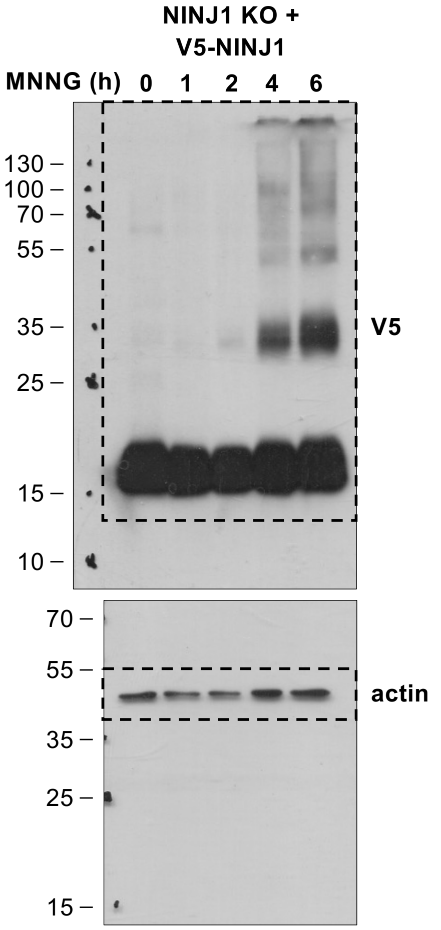

SFig. 5B

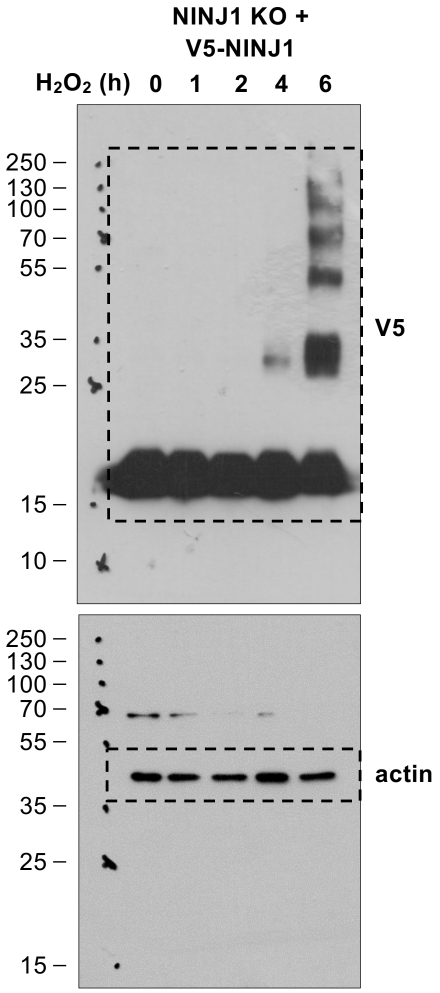

SFig. 5C

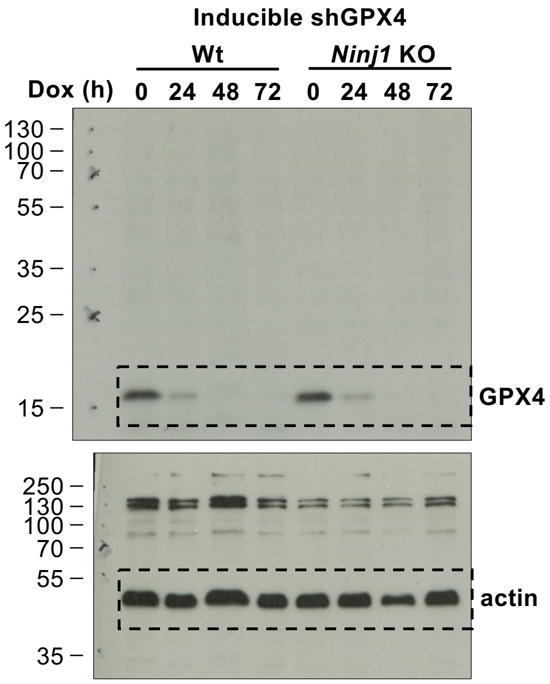

SFig. 6C

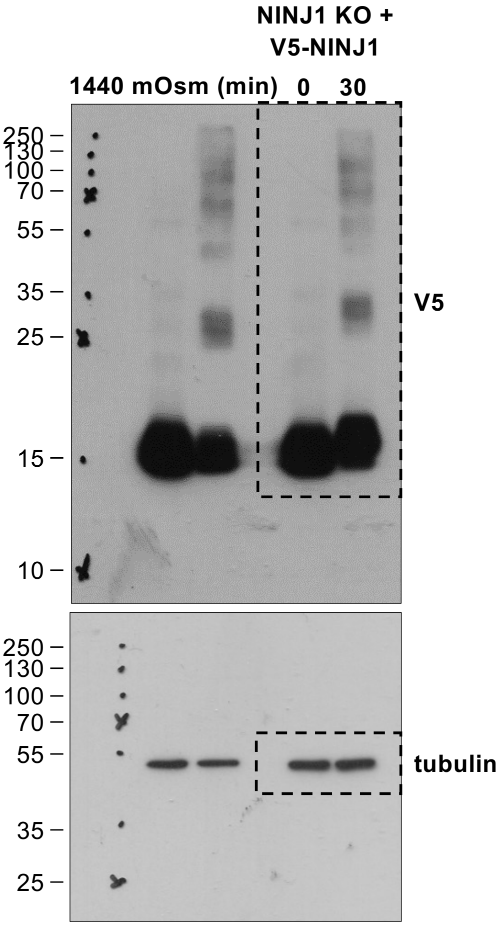

Supplement: Supplementary file 3 — Uncropped blots [file 41419_2023_6284_MOESM3_ESM.pdf]
