# Supplementary material for: NINJ1 is activated by cell swelling to regulate plasma membrane permeabilization during regulated necrosis
Source: Cell Death Dis. 2023 Nov 18;14(11):755. doi: 10.1038/s41419-023-06284-z (PMC10657445; doi:10.1038/s41419-023-06284-z)
Supplement: Supplementary file 4 — Author list agreement [file 41419_2023_6284_MOESM4_ESM.pdf]

From Yves Dondelinger <yvesdondelinger@hotmail.com>
To Me
Subject **Re: CDDIS-23-2034RR Initial Quality Check**

Reply Forward Archive Junk Delete More

17:31

Hello Mathieu,

I agree with the changed author list.

Best wishes,
Yves

Sent from Outlook for Android

From: Mathieu Bertrand <mathieu.bertrand@irc.vib-ugent.be>
Sent: Friday, November 3, 2023 11:17:12 am
To: Yves Dondelinger <yvesdondelinger@hotmail.com>; peter.vandenabeele@irc.vib-ugent.be <peter.vandenabeele@irc.vib-ugent.be>; Dario Priem <dario.priem@irc.vib-UGent.be>; Tom.Delanghe@irc.vib-Ugent.be <Tom.Delanghe@irc.vib-Ugent.be>; Jon.Huyghe@irc.vib-UGent.be <Jon.Huyghe@irc.vib-ugent.be>
Subject: Fwd: CDDIS-23-2034RR Initial Quality Check

Dear all,

In order to proceed further with our accepted manuscript, CDDis is asking that all authors confirm their agreement with the change in the author list made between the initial submission and accepted manuscript. The addition of the authors is justified by their contribution to the generation of the revised (and accepted) version.

From:

Yves Dondelinger[1,2,*], Peter Vandenabeele[1,2] and Mathieu JM Bertrand[1,2,*]

to

Yves Dondelinger[1,2,*], Dario Priem[1,2], Jon Huyghe[1,2], Tom Delanghe[1,2], Peter Vandenabeele[1,2] and Mathieu JM Bertrand[1,2,*]

Could I therefore ask you to reply to this email mentioning your agreement?

Thank you.

Best,

Mathieu

Inbox - mathieu.bertrand@irc.vib-ugent | Re: Fwd: CDDIS-23-2034RR Initial Q

From Dario VIB <dario.priem@irc.vib-ugent.be>
To Me, Yves Dondelinger <yvesdondelinger@hotmail.com>, Tom.Delanghe@irc.vib-Ugent.be <Tom.Delanghe@irc.vib-Ugent.be>, Jon.Huyghe@irc.vib-UGent.be <Jon.Huyghe@irc.vib-ugent.be>, Peter Vandenabeele <Peter.Vandenabeele@irc.vib-ugent.be>
Subject **Re: Fwd: CDDIS-23-2034RR Initial Quality Check**

Reply Reply All Forward Archive Junk Delete More

19:31

Dear,

I agree with the author list.

Kind regards,

--

Dario Priem, PhD
Postdoctoral Scientist - Bertrand Group

VIB-UGent Center for Inflammation Research
Technologiepark 71, 9052 Zwijnaarde (Ghent), Belgium
Phone: (+32)-9-33 13713

On 3 Nov 2023 at 18:31 +0100, Peter Vandenabeele <Peter.Vandenabeele@irc.vib-ugent.be>, wrote:

> Dear Mathieu,
>
> I agree with the author adaptations.
>
> Kind regards,
>
> Peter
>
> On 03-11-2023 16:17, Mathieu Bertrand wrote:
>
>> Dear all,
>>
>> In order to proceed further with our accepted manuscript, CDDis is asking that all authors confirm their agreement with the change in the author list made between the initial submission and accepted manuscript. The addition of the authors is justified by their contribution to the generation of the revised (and accepted) version.
>>
>> From:
>>
>> Yves Dondelinger[1,2,*], Peter Vandenabeele[1,2] and Mathieu JM Bertrand[1,2,*]

Inbox - mathieu.bertrand@irc.vib-ugent... | Re: Fwd: CDDIS-23-2034RR Initial Q...

Reply | Reply All | Forward | Archive | Junk | Delete | More

From Jon Huyghe <jon.huyghe@irc.vib-ugent.be>

To Dario VIB <dario.priem@irc.vib-ugent.be> 03/11/2023, 20:43

Cc Me, Yves Dondelinger <yvesdondelinger@hotmail.com>, Tom.Delanghe@irc.vib-Ugent.be <Tom.Delanghe@irc.vib-ugent.be>, Peter Vandenabeele <Peter.Vandenabeele@irc.vib-ugent.be>

Subject **Re: Fwd: CDDIS-23-2034RR Initial Quality Check**

Dear,

I also agree with the author list.

Kind regards,

Jon

On 2023-11-03 19:31, Dario VIB wrote:

> Dear,
>
> I agree with the author list.
>
> Kind regards,
>
> --
>
> **Dario Priem, PhD**
> Postdoctoral Scientist - Bertrand Group
>
> **VIB-UGent Center for Inflammation Research**
> Technologiepark 71, 9052 Zwijnaarde (Ghent), Belgium
> Phone: (+32)-9-33 13713
>
> On 3 Nov 2023 at 18:31 +0100, Peter Vandenabeele <Peter.Vandenabeele@irc.vib-ugent.be>, wrote:
>
>> Dear Mathieu,
>>
>> I agree with the author adaptations.
>>
>> Kind regards,
>>
>> Peter
>>
>> On 03-11-2023 16:17, Mathieu Bertrand wrote:
>>
>>> Dear all,
>>>
>>> In order to proceed further with our accepted manuscript, CDDis is asking that all authors confirm their agreement with the change in the author list made between the initial submission and accepted manuscript. The addition of the authors is justified by their contribution to the generation of the revised (and accepted) version.

Inbox - mathieu.bertrand@irc.vib-ugent... | Re: Fwd: CDDIS-23-2034RR Initial Q...

Reply | Reply All | Forward | Archive | Junk | Delete | More

From Tom Delanghe <tom.delanghe@irc.vib-ugent.be>

To Me 04/11/2023, 15:48

Cc Yves Dondelinger <yvesdondelinger@hotmail.com>, peter.vandenabeele@irc.vib-ugent.be, Dario Priem <dario.priem@irc.vib-ugent.be>, Jon.Huyghe@irc.vib-UGent.be <Jon.Huyghe@irc.vib-ugent.be>

Subject **Re: Fwd: CDDIS-23-2034RR Initial Quality Check**

I agree with the author list

On 2023-11-03 16:17, Mathieu Bertrand wrote:

> Dear all,
>
> In order to proceed further with our accepted manuscript, CDDis is asking that all authors confirm their agreement with the change in the author list made between the initial submission and accepted manuscript. The addition of the authors is justified by their contribution to the generation of the revised (and accepted) version.
>
> From:
>
> Yves Dondelinger[1,2,*], Peter Vandenabeele[1,2] and Mathieu JM Bertrand[1,2,*]
>
> to
>
> Yves Dondelinger[1,2,*], Dario Priem[1,2], Jon Huyghe[1,2], Tom Delanghe[1,2], Peter Vandenabeele[1,2] and Mathieu JM Bertrand[1,2,*]
>
> Could I therefore ask you to reply to this email mentioning your agreement?
>
> Thank you.
>
> Best,
>
> Mathieu
